# Gut microbiota orchestrates bone homeostasis: a multi-pathway network from intestine to skeleton

**DOI:** 10.3389/fendo.2026.1767726

**Published:** 2026-04-30

**Authors:** Yanlong Gong, Xin Ma, Lele Wang, Pengwei Zhang, Tingting Liu, Yuanzhen Li, Wantao Dong

**Affiliations:** 1 Intervertebral Disc Center, Affiliated Hospital of Gansu University of Chinese Medicine, Lanzhou, China; 2 School of Clinical Chinese Medicine, Gansu University of Chinese Medicine, Lanzhou, China; 3 The Second Clinical Medical College, Henan University of Chinese Medicine, Zhengzhou, China; 4 Department of Sports Medicine, Affiliated Hospital of Gansu University of Chinese Medicine, Lanzhou, China

**Keywords:** bone metabolism, gut microbiota, gut-bone axis, immune regulation, osteoporosis, probiotics, SCFAs

## Abstract

Osteoporosis (OP), a widespread metabolic bone condition characterized by diminished bone mass and compromised microarchitecture, poses a significant global health challenge. The gut microbiota (GM) regulates bone homeostasis through the “gut-bone axis,” and this review consolidates its diverse mechanisms. GM-derived metabolites directly/indirectly modulate osteoclast/osteoblast activity. GM also regulates systemic immunity to influence the RANKL/OPG pathway and mediates endocrine signals. Furthermore, it modulates intestinal barrier integrity to facilitate mineral/vitamin absorption and interacts with the nervous system to form the “microbiota-gut-brain-bone” axis. GM imbalance, resulting from factors such as aging, hormonal shifts, or dietary habits, promotes the progression of OP through the perturbation of these networks. This review evaluates the therapeutic potential of GM-targeted interventions, including probiotics, prebiotics, and fecal microbiota transplantation, and underscores the GM as a pivotal therapeutic target, emphasizing that future therapeutic strategies for OP must incorporate the interconnected GM-bone axis for efficacious prevention and treatment.

## 1 Introduction

Microorganisms derived from both the maternal body and the external environment begin to colonize the intestine at birth, progressively forming a structurally stable community known as the GM (1). The GM has a gene pool that is 150 times more extensive than that of the human genome. Co-evolving with humans, it affects the host in several ways, prompting some researchers to regard the GM as a “forgotten human organ” (2). There exists an inseparable and mutualistic interaction between the GM and the host’s physiology, directly impacting individual health. Under homeostatic conditions, the microbiota obtains vital nutrients and energy from the host intestine, simultaneously aiding the host in food digestion, generating metabolites, and providing nutrients. It also strengthens the intestinal barrier to protect against invasive microorganisms and shield the host from illnesses. Furthermore, the GM is crucial in regulating host metabolic processes and immunological functionality (3). Recent findings underscore the vital significance of the GM in human health and disease, generating considerable interest in elucidating these microbial processes and their mechanisms of influencing host physiology (4).

In 1976, esteemed researcher Genant et al. (5) first documented aberrant bone metabolism in subjects with inflammatory bowel disease (IBD), observing diminished bone mass, particularly among adolescents. The GM influences bone health via various mechanisms; its immunomodulatory effects, among others, act as a vital link between bacteria and bone metabolism—a paradigm commonly referred to as the “gut-bone axis” (6). OP is a prevalent age-related condition marked by diminished bone mass and deterioration of bone tissue structure, leading to heightened bone fragility and elevated risk of fractures (7). remodeling Fundamentally, OP results from dysregulated bone remodeling. Despite the availability of various medications for managing OP, a significant number of patients remain untreated due to financial constraints and the adverse side effects associated with currently available pharmacological agents (8). In healthy adults, bone remodeling is balanced through the differentiation, activation, and apoptosis of bone-forming osteoblasts and bone-resorbing osteoclasts. In aging, especially among postmenopausal women (PMW), changes in the GM may stimulate or regulate the immune system. Under inflammatory conditions, bone-resorptive cytokines, enhanced through microbiota-dependent T-cell pathways, can increase osteoclastic activity (9). Increasing data suggest that interactions between environmental factors and the GM elevate the risk of OP, with aging, medicine, nutrition, and environment serving as prevalent influences on the GM (10).

**Figure 1:**
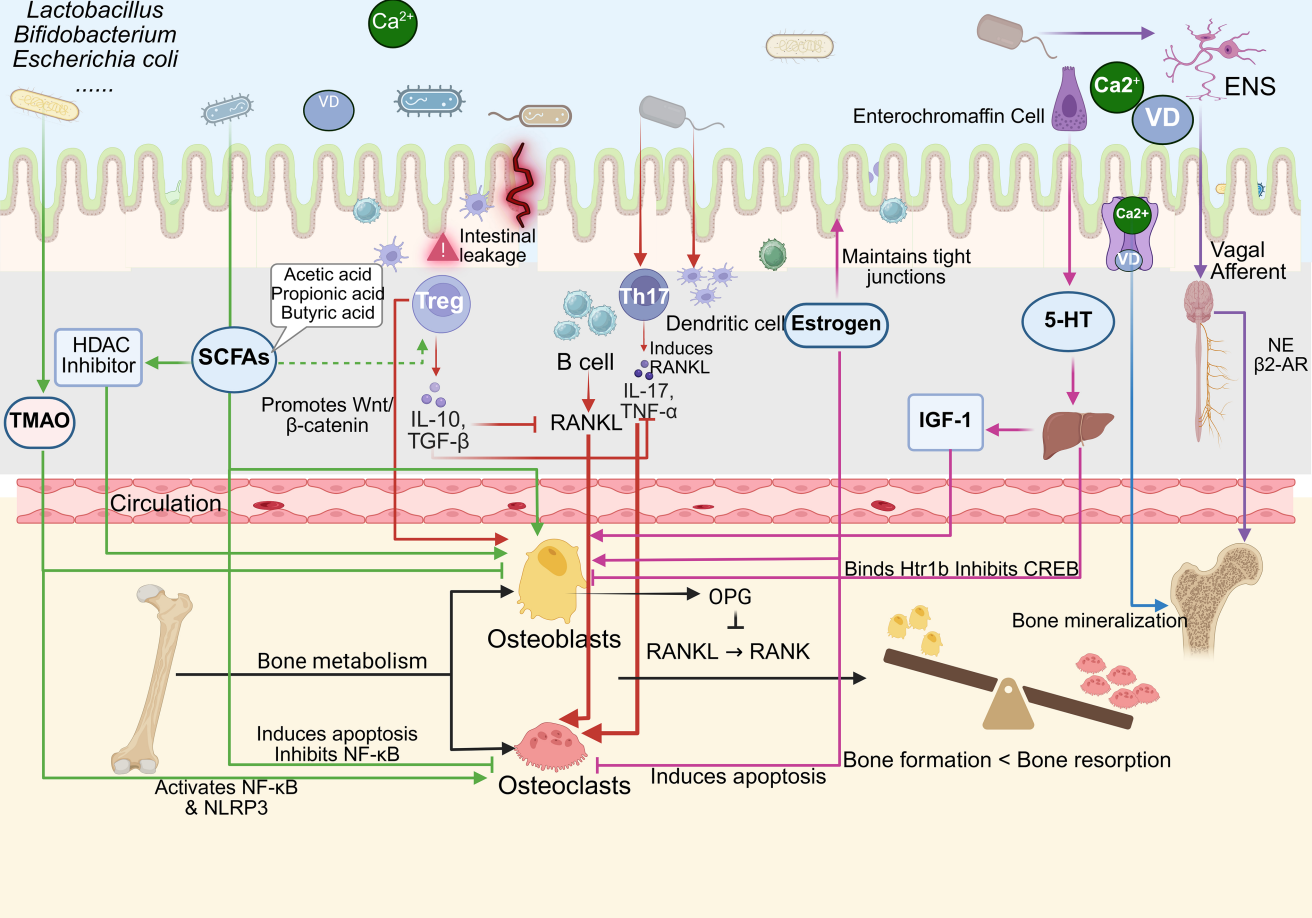
The method by which GM modulates bone metabolism via immune regulation. Created in Biorender.com.

Recent research has progressively suggested that damaged or dysregulated GM may result in inadequate bone development, skeletal disorders, and abnormalities in other systems. Contemporary research frequently emphasizes discrete elements such as microbial metabolite synthesis, host metabolic processes, intestinal barrier function, immunological modulation, or pharmacokinetics. Nevertheless, the GM constitutes a huge and complex ecosystem, and there remains a deficiency of comprehensive research assessing its total functional impact on OP. Research on altering the GM for the prevention and treatment of OP is not only justified but may also provide innovative approaches for OP management. This paper summarizes a thorough examination of the influence of GM on OP, as depicted in **Figure 1**.

## 2 Literature search strategy

To ensure a comprehensive and rigorous synthesis of the current knowledge regarding the gut-bone axis, a thorough literature search was conducted. Although this is a narrative review, we adopted a systematic approach for literature retrieval to enhance reproducibility.

We searched primary electronic databases, including PubMed, Web of Science, and Google Scholar, for articles published from their inception up to February 2026. The search strategy utilized combinations of the following Medical Subject Headings (MeSH) and text words: (“gut microbiota” OR “gastrointestinal microbiome” OR “dysbiosis”) AND (“osteoporosis” OR “bone metabolism” OR “bone mineral density” OR “osteoclast” OR “osteoblast”) AND (“short-chain fatty acids” OR “immune regulation” OR “gut-bone axis” OR “mechanostat”).

The inclusion criteria were (1): original research articles, clinical trials, meta-analyses, and comprehensive reviews relevant to the topic; (2) studies investigating the mechanistic or clinical links between the gut microbiome and bone health; and (3) articles published in English. The exclusion criteria were: (1) abstracts without available full texts; (2) case reports or non-peer-reviewed preprints; and (3) studies focusing on bone diseases unrelated to metabolic or microbiota-driven pathways (e.g., traumatic fractures without a systemic context). Two authors independently screened the titles and abstracts, followed by a full-text review of relevant articles to extract key mechanisms and clinical findings discussed in this review.

## 3 Impact of GM on intestinal function

The GM is crucial to the development, functioning, and homeostasis of the host intestine. It substantially aids in the structural development of the digestive tract. Studies have demonstrated that germ-free animals display cecal enlargement, reduced intestinal surface area, and gastrointestinal dysregulation (11, 12). The reduction in intestinal surface area is due to decreased cell renewal and lower leukocyte infiltration, resulting in thinner intestinal villi. Moreover, germ-free animals exhibit a diminished capillary network in the villi, which hinders nutritional absorption efficacy. Nutrients such as calcium and vitamins, taken by the colon, significantly influence bone metabolism. The intestinal mucosa, being the most direct interface between gut microbes and host tissues (13), enables numerous biological processes—including metabolite processing and immunological modulation—that are pertinent to bone metabolism.

### 3.1 Intestinal mucosa

As the innermost layer of the intestinal wall, the mucosa is divided into the small and large intestinal segments. The mucosa of the small intestine exhibits many circular folds and villi formations. Its epithelial layer mostly comprises absorptive cells interspersed with goblet cells that generate sialic mucus. The lamina propria has an abundance of blood arteries, nerves, and lymphoid tissue, while the muscularis mucosa consists of 3 to 10 layers of smooth muscle cells. This structure functions as the principal site where GMs participate in the regulation of intestinal function. As the primary defense against pathogenic and non-pathogenic bacteria, the intestinal mucosal barrier—composed of epithelial cells—is shielded by a host-secreted mucous layer.

The equilibrium of the gut microbial community plays a pivotal role in sustaining homeostasis—or responding to any disruptions within and beyond the intestinal mucosa (14). Aline Ignacio et al. found that the GM is a crucial regulator of the activation, heterogeneity, and function of eosinophils in the small intestine. Their findings demonstrate that eosinophils are predominantly located in the villi and crypt sections of the intestinal mucosa, while being lacking in the muscularis mucosa. Eosinophil insufficiency results in morphological and physiological changes in the small intestine after microbial colonization, characterized by diminished villi, compromised barrier integrity, and decreased motility (15). Moreover, a study conducted by Bo Li et al. demonstrated that puerarin mitigates osteoporosis in ovariectomized (OVX) rats chiefly by modulating the GM. This intervention facilitates the release of short-chain fatty acids (SCFAs), such as butyrate and valerate, which aid in restoring intestinal mucosal integrity and maintaining mucosal homeostasis. Ultimately, the rectification of gut microbial dysbiosis contributes to the amelioration of OP (16).

### 3.2 Intestinal epithelial cells

The intestinal epithelium, covered by intestinal epithelial cells (IECs), represents the largest mucosal surface in the human body and serves as a central interface for host-microbiota interactions. While digesting and absorbing nutrients to meet the host’s energy demands, the intestinal epithelium is consistently exposed to harsh mechanical and chemical stresses (17). IECs exhibit one of the highest cellular turnover rates in the body. They constitute the primary site for nutrient and fluid absorption, yet must also maintain a tight barrier to prevent pathogen invasion—processes that are tightly regulated (18). The monolayer constituted by IECs serves as a mediator between the intestinal lumen and the internal environment, as well as a vital element of the body’s immune barrier (19). Excessive proliferation of IECs can result in extensive epithelial erosion, a characteristic of some intestinal diseases (20). As essential regulators of barrier function and immune homeostasis, IECs are influenced by the GM and regulate bone metabolism through many routes (21).

Bone metabolism, resorption, and balance are critical for skeletal health (22). Among the functions of Ion Transient Receptor Potential Cation Channel Subfamily V Member 6 (TRPV6) is the key mechanism on IECs that allows active Ca²^+^ entry. Although the overall extent of homology with TRPV5 is quite close, it is still necessary to distinguish the separate localizations of both proteins; TRPV6 mediates the major pathway of calcium transcellular absorption in the gastrointestinal tract, while TRPV5 is primarily located in the renal nephron at the distal tubules and collecting ducts, which are the sites of renal calcium reabsorption. In turn, intestinal TRPV6 is under the tight regulation of the gut microbes, calcitriol, estrogen, and dietary calcium. Although TRPV6 is a key mediator of intestinal calcium absorption in humans, significant Ca²^+^ uptake still occurs in TRPV6 ^-^/^-^ mice, suggesting compensatory mechanisms via other channels or molecules (23). Calcium extrusion from IECs is mediated by two primary proteins: plasma membrane calcium ATPase 1 (PMCA1) and sodium-calcium exchanger 1 (NCX1). Protein 4.1R may modulate PMCA1b function; Liu et al. (24) found that 4.1R co-localizes with PMCA1b, and 4.1R mutant animals display compromised intestinal calcium absorption alongside diminished PMCA1b expression. PMCA1b is the primary protein responsible for Ca²^+^ extrusion from IECs (25), while NCX1 contributes approximately 20% of calcium transport from the intestine into the circulation (26).

The functionality and calcium transport mechanism of IECs are intricately linked to Vitamin D (VitD), and a shortage in VitD has a considerable detrimental effect on bone health. VitD deficiency is linked to diminished bone mineralization and heightened cortical bone loss, which exacerbates the development and progression of OP in the elderly, hence increasing bone fragility (27). It may also result in diminished muscle mass and strength (28), hence increasing the risk of falls and associated fractures (29). The intestinal absorption of VitD from dietary sources and supplements plays a pivotal role in maintaining adequate VitD levels, particularly in cases of deficiency (30). The forms of VitD obtained from the intestine include ergocalciferol (vitamin D_2_) and cholecalciferol (vitamin D_3_), both of which can be acquired from various diets, though fortified goods and supplements are significant sources (31, 32). Specific probiotic strains, including *Lactobacillus rhamnosus* GG (LGG), affect the absorption of cholecalciferol by modulating the expression of VitD transporters in IECs. The augmented expression of Niemann-Pick C1-like 1 (NPC1L1) and cluster determinant 36 (CD36) in the brush border membrane of jejunal and ileal epithelial cells (33), coupled with diminished expression of scavenger receptor class B type I (SR-BI) (34), ultimately enhances the intestinal absorption of cholecalciferol, potentially benefiting bone health in age-related OP. The GM can modify intestinal VitD metabolism, and probiotic administration has been demonstrated to affect circulating VitD metabolites (35). Technically, a screening test of a blood sample for serum VitD is the main proxy measure of total VitD stores and nutritional status. Nonetheless, 1,25(OH)_2_D, the biologically active form, is tightly regulated by parathyroid hormone (PTH), calcium, and phosphate homeostasis; consequently, the total body levels do not completely express the body’s VitD stores (36).

Lastly, it is the active metabolites of VitD that play a significant role in this microbiome interaction. A cross-sectional study was conducted by Robert L. Thomas et al. (37) in 567 older men, using LC-MS/MS to measure serum VitD metabolites and 16S rRNA gene sequencing to define fecal microbial composition. The findings indicated that serum 1,25(OH)_2_D levels accounted for 5% of the variance in alpha diversity (Faith’s Phylogenetic Diversity). In unweighted UniFrac beta-diversity analysis, 1,25(OH)_2_D emerged as the most significant predictor, accounting for 2% of the variation. Men exhibiting elevated levels and activation rates of active VitD—not 25(OH)D—demonstrated a link between active VitD and overall gut microbial diversity. Twelve particular bacterial taxa exhibited greater abundance in persons with elevated active VitD, the majority of which are butyrate-producing bacteria. Butyrate, a beneficial SCFA, maintains intestinal integrity, modulates epithelial function, and regulates gut immunity and microenvironment. Moreover, a study conducted by Dapeng Jin et al. (38)revealed bidirectional signaling between gut microorganisms and colonic epithelial cells, suggesting that intact VitD signaling is essential for maintaining a healthy gut microbial community. Mice with impaired VitD metabolism were found to exhibit gut dysbiosis. These findings provide novel insights into the prevention and treatment of OP.

### 3.3 Intestinal permeability

The intestinal barrier and intestinal permeability are essential factors in health and disease (39). As a vast mucosal surface constantly exposed to billions of gut bacteria, the intestinal barrier constitutes the body’s most extensive immune system. The intact intestinal barrier serves a dual function: it defends the body against harmful bacteria and toxins while facilitating the absorption of essential fluids and minerals. This dual function is accomplished by a complex anatomical and functional structure, characterized by its operational condition known as “intestinal permeability” (40). Various variables can modify intestinal permeability (41), including changes in the GM, as well as alterations in the mucus layer and epithelial damage—all of which collectively enable the translocation of luminal materials into the intestinal wall. Lifestyle and dietary elements, such as alcohol consumption and a nutrient-deficient diet, may potentially enhance permeability (42). Modified intestinal permeability resulting from microbial dysbiosis may impact the absorption of minerals and other nutrients, thereby affecting bone health (43).

The GM and pathogens frequently enhance barrier permeability by modifying the construction and function of tight junctions (TJ)—which seal the paracellular space—via the production of proteases that break TJ proteins or modify the cytoskeleton (44). In addition, inflammatory cytokines such as TNFα and IFNγ have been demonstrated to increase intestinal permeability (45), while probiotics and commensal bacteria can mitigate this inflammation and rehabilitate epithelial function in human gut models (43). In a study by Zheng Zhang et al. (46), the effects of prebiotics on obesity-related bone loss were investigated. In this study, subjects subjected to a high-fat diet (HFD) to induce obesity were administered fructo-oligosaccharides (FOS) and galacto-oligosaccharides (GOS). The findings indicated that long-term HFD led to reduced bone mass, gut dysbiosis, intestinal permeability, and systemic inflammation. The administration of FOS/GOS markedly enhanced gut microbial biodiversity as indicated by an increased *Firmicutes*-to-*Bacteroidetes* ratio and restored alpha diversity alongside increased concentrations of SCFAs—including acetate, propionate, and butyrate. This intervention reversed high intestinal permeability and levels of inflammatory cytokines (including TNFα, IL-6, and IL-17), ultimately protecting against HFD-induced osteopenia. Intestinal permeability was evaluated by means of FITC-dextran. The study found that FOS/GOS supplementation inhibited the dysregulated expression of intestinal epithelial gap junction proteins—including Claudin-1, Claudin-15, Zonula occludens-1 (ZO-1), and Junctional adhesion molecule-A—thereby mitigating barrier damage and reversing HFD-induced hyperpermeability. These findings suggest that prebiotic modulation of the GM offers a promising strategy for preventing and treating obesity-related OP.

The intestine facilitates material exchange with luminal contents through specialized pathways—notably the paracellular pathway, which typically permits the passage of molecules larger than 15 Å (approximately 3.5 kDa) (47, 48). Enhanced intestinal permeability expands the spectrum of molecules and potential antigens capable of traversing the intestinal barrier. These substances can reach the submucosal layer, contributing to intestinal pathologies and systemic pro-inflammatory responses (49–51). Compromise of tight junction structures between the intestinal lumen and the submucosa may allow immune cells in the subepithelial compartment to produce osteoclastogenic cytokines, which can subsequently enter systemic circulation and disrupt bone homeostasis (52). Recent research demonstrates that decreased estrogen levels enhance intestinal permeability, undermine barrier integrity, promote bacterial translocation, and increase inflammatory markers, thereby promoting the synthesis of osteoclastogenic cytokines (53, 54). Increased intestinal permeability and a weakened gut barrier lead to higher levels of cytokines, potentially contributing to OP. This mechanistic insight uncovers novel avenues for the prophylaxis and management of estrogen deficiency-associated OP. Furthermore, the GM can affect intestinal function—and consequently bone metabolism—by modulating intraepithelial lymphocytes (55) and various immune cells (56) —a process that may indirectly regulate intestinal permeability by preserving epithelial barrier integrity, thereby indirectly supporting bone metabolism.

## 4 Alterations in the structural and functional characteristics of the GM

The GM is intricately linked to health in the aged, as it may influence age-related alterations in innate immunity, sarcopenia, bone metabolism, and cognitive function—all of which contribute to frailty in older persons. Research demonstrates that the GM of older individuals differs from that of younger adults. Rather than undergoing abrupt shifts at a specific age threshold, the GM in the elderly evolves gradually throughout time (57). The structural composition and functional capacity of the GM are essential for preserving host tissue integrity and are associated with age-related degenerative diseases (58). Increasing data indicate that intestinal dysbiosis is associated with senile OP (59–61). For example, Das M et al. found that the ratios of *Escherichia*/*Shigella* and *Escherichia*/*Vermella* were higher in persons with osteopenia than in those with OP. In contrast, higher abundances of *Actinomyces*, *Eggerthella*, *Clostridium* Cluster XlVa, and *Lactobacillus* were observed in OP patients relative to those with normal bone mineral density (BMD) (62).

Katharina E. Scholz-Ahrens et al. (63) define gut dysbiosis as a deviation in the microbial ecology resulting in a diminished or skewed composition linked to illness and bone loss. Age-related metabolic dysregulation is characterized by diminished nutritional assimilation (64), genetic instability (65), compromised intercellular communication (66), and modifications in the GM (67). In a study using the senescence-accelerated mouse model SAMP6, Kenichi Tanabe et al. (68) administered FOS and glucomannan as treatments. The findings revealed a significant increase in BMD in both the FOS and glucomannan intervention groups when compared with the control cohort. In the FOS group, cecal contents exhibited substantial elevations in *Lactobacillus* and *Bacteroides* abundances, whereas the glucomannan group showed a rise in *Clostridium* levels. Urinary deoxypyridinoline concentrations and high-sensitivity C-reactive protein (hs-CRP) levels were notably decreased in the FOS and glucomannan groups relative to control subjects. This work illustrates that dietary FOS and glucomannan alter the GM, reduce systemic inflammation related to aging, and decrease bone resorption.

Studies indicate that the prevalence of four principal microbial phyla—Firmicutes, Actinobacteria, Bacteroidetes, and Proteobacteria—correlates with host bone mass (69, 70). Research conducted by Ling et al. (71)and Rettedal et al. (72)utilizing 16S rRNA sequencing demonstrated disparities in the GM between OP patients with osteoporotic fractures and healthy controls. *Blautia*, *Actinobacillus*, *Oscillospira*, *Bacteroides*, and *Phascolarctobacterium* exhibited a positive correlation with OP, while *Veillonellaceae*, *Collinsella*, and *Ruminococcaceae* demonstrated a negative association with bone loss. In a study investigating gut microbiome characteristics and BMD changes in older Chinese individuals, Yangyang Wang et al. (73) noted more microbial operational taxonomic unit (OTU) richness in women compared to males. At the order level, the relative abundance of Clostridiales was positively correlated with BMD. PMW, experiencing a notable reduction in sex hormones, exhibited increased vulnerability to GM-related alterations in BMD. Metagenomic sequencing indicated a somewhat greater quantity of microbial genes in women compared to men. Zhongxiang Wang et al. (53) documented a notable decrease in *Prevotella* abundance in postmenopausal osteoporosis patients (PMOP), demonstrating protective effects on bone mass, likely through modulation of intestinal permeability and inhibition of osteoclast activity via suppression of osteoclastogenic cytokines. Estrogen levels influence immunological response and microbial composition. Decreasing estrogen levels diminish microbial diversity, notably resulting in a reduction of *Firmicutes* such as *Clostridium* (74). Additional research demonstrates a significant link between postmenopausal urine estrogen metabolites and both microbial diversity and the relative abundance of Clostridium and Bacillus in feces (75). Advancing age and diminished gastrointestinal function are risk factors for OP (76). As individuals age, there is an increase in pathogenic and putrefactive bacteria (e.g., *Proteus*), while helpful bacteria such as *Lactobacillus* and *Bifidobacterium* diminish. These compositional changes indirectly elicit immunological responses, promote bone resorption, and contribute to OP (77). These outcomes underscore the biological significance of a stable gut microbial composition in OP and offer fresh insights into the interplay between gut bacteria and skeletal health.

## 5 Impact of gut microbial metabolites on bone metabolism

Under physiological settings, the GM participates in intricate and dynamic metabolic activities within the human intestine. These mechanisms supply energy and nutrients for their own growth and reproduction while producing many metabolites that infiltrate the host system (82, 83). The main substrates for gut microbial metabolism include unprocessed or partially digested food elements and endogenous mucus produced by IECs. Through microbial action, these substrates are converted into various metabolites that may be advantageous or harmful to the host, including SCFAs, choline metabolites, vitamins, lipids, phenolic compounds, cortisol, genetic materials, and phytoestrogens (84–86). The GM has a crucial regulatory role in material metabolism, growth and development, as well as bone metabolism and homeostasis (87). A multitude of microbial metabolites influence bone remodeling. For instance, even moderate amounts of folate can increase peripheral serum homocysteine, potentially inducing osteoblast apoptosis and stimulating osteoclast formation by promoting intracellular reactive oxygen species (ROS), hence contributing to OP (88, 89). Cortisol can impair bone microstructure via multiple mechanisms, including modifying bone cell function, regulating inflammatory cytokines, and influencing calcium metabolism (69), among others.

### 5.1 Short-chain fatty acids

SCFAs, as final products of intestinal microbiota fermentation, might influence metabolic responses in numerous peripheral organs following absorption from the gut lumen (90). The human GM, comprising approximately 10¹^4^ cells per gram, is primarily constituted of anaerobic bacteria such as *Bifidobacterium* and *Bacteroides*, which represent over 98% of the total population (91). SCFAs are primarily synthesized in the cecum and proximal colon. The primary SCFAs are acetate, propionate, and butyrate, with supplementary variants such as isobutyrate, isovalerate, and valerate (92). Acetate and propionate are mostly generated by the phylum *Bacteroidetes* (93), while butyrate is chiefly synthesized by *Firmicutes* (94).

**Figure 2:**
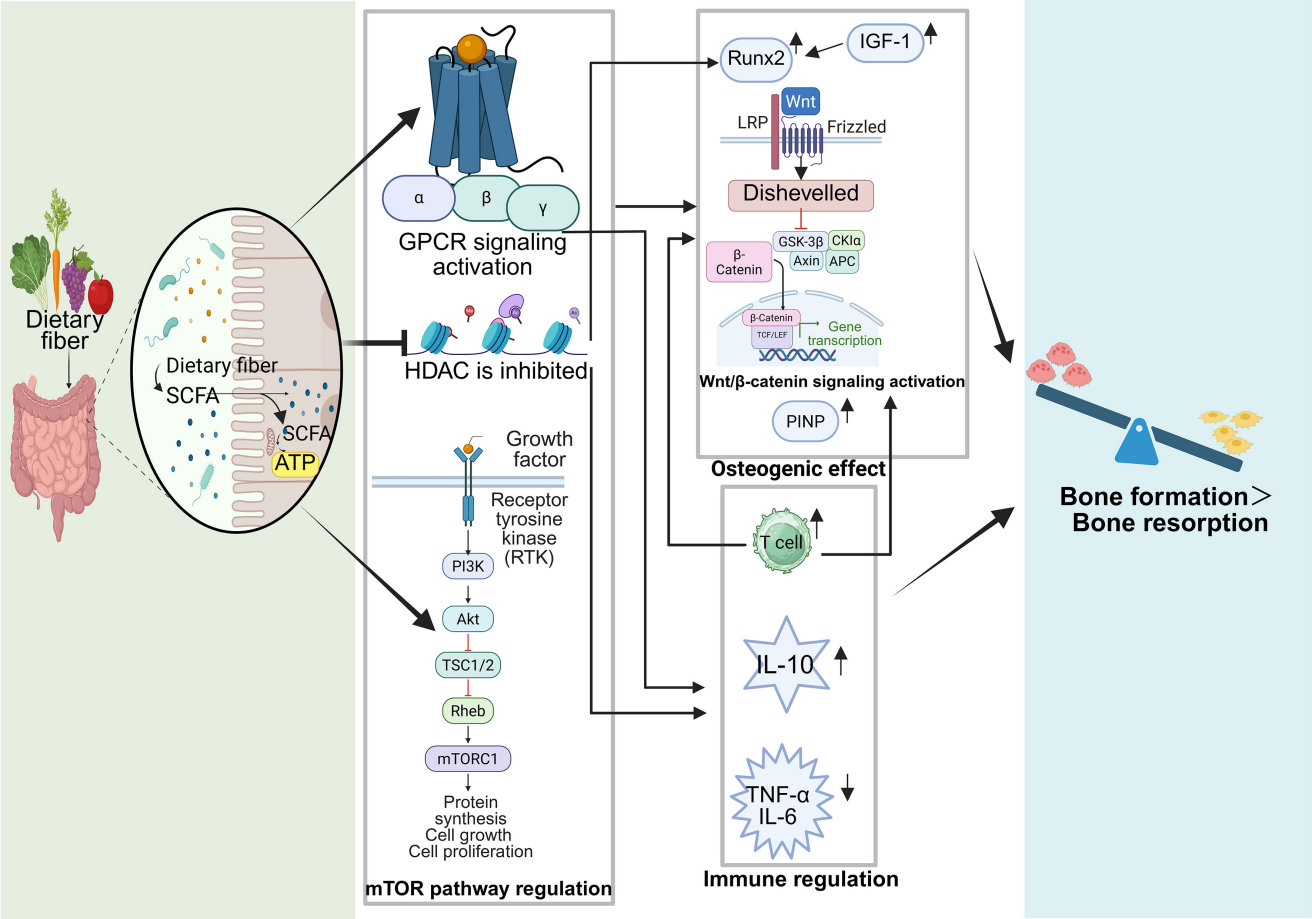
The multifaceted mechanisms of SCFAs in regulating bone metabolism. Created in Biorender.com.

SCFAs are vital regulators of human metabolism and are essential to maintaining a healthy and functionally intact intestinal mucosa (95). They serve as the principal energy substrate for IECs, directly influencing their growth and differentiation (96). As key mediators within the gut-bone signaling axis, SCFAs can affect bone metabolism by modulating osteoclasts and osteoblasts, either directly or indirectly through immune regulatory mechanisms. These interactions modify the equilibrium between bone creation and resorption, thereby influencing the development of OP (97, 98). Recent studies demonstrate that SCFAs affect bone metabolism via the processes outlined (**Figure 2**): ①Intestinal Epithelial Function and Mineral Absorption: SCFAs act on IECs to promote their development and differentiation, hence affecting the mechanical, chemical, immune, and biological barriers of the intestinal mucosa, regulating electrolyte exchange (such as between mineral ions and hydrogen ions), lowering luminal pH, and directly improving the absorption of minerals specially calcium (99, 100);②Systemic Signaling via G protein-coupled receptors (GPCRs): Upon entering systemic circulation, SCFAs interact with GPCRs, influencing metabolism and function in peripheral tissues, including adipose tissue, skeletal muscle, bone, and liver (101);③Epigenetic Regulation: SCFAs block histone deacetylases (HDACs), therefore contributing to the epigenetic regulation of gene expression, which may influence bone metabolism (102, 103);④Modulation of Inflammatory Cytokines: SCFAs can modulate the production of inflammatory factors, therefore affecting bone remodeling (54);⑤Immune Cell Regulation: SCFAs facilitate the development of regulatory T (Treg) cells and trigger death in macrophages and neutrophils. This may aid in preventing excessive immune activation and indirectly affect bone metabolism (104).

Although bacteria that produce SCFAs and SCFAs themselves confer advantages to human health, it is uncertain whether these effects are solely due to SCFAs alone or result from synergistic actions with other metabolites produced by these bacteria. Propionate and butyrate (C4) are capable of triggering metabolic reprogramming within osteoclasts, promoting glycolysis while suppressing oxidative phosphorylation. This substantially inhibits the expression of two pivotal osteoclastogenic genes, TRAF6 and NFATc1, resulting in a considerable decrease in serum levels of bone resorption markers (105). Butyrate enhances the quantity of regulatory T cells (Tregs) in the gastrointestinal tract and bone marrow (106), upregulates the VitD receptor (VDR), and alters proteins associated with calcium absorption, therefore affecting bone metabolism (107). SCFAs inhibit HDACs, increase acetylation of p70S6 kinase and phosphorylation of rS6, and augment the proportions of Th1, Th17, and IL-10-producing T cells, thus influencing the mTOR pathway (108). Th1 and Th2 cells can suppress osteoclast activation by classical cytokines such as IFN-γ and IL-4 (109). Nonetheless, IFN-γ may also indirectly facilitate bone resorption by increasing RANKL and TNF (110), indicating that SCFAs exert a dual regulatory role on osteoclast activity. A study by Duscha et al. (111) revealed that c(PA) supplementation not only increased Treg populations and conferred immunomodulatory advantages in patients with multiple sclerosis (MS) but also diminished serum β-Cross Laps levels and increased osteocalcin levels, indicating a protective effect against OP in MS patients. Feiyan Zhao et al. (78) found that a three-month co-administration of *Bifidobacterium lactis* Probio-M8 with calcium and calcitriol improved bone metabolism in patients, as evidenced by decreased levels of bone turnover markers such as alkaline phosphatase (ALP) and osteocalcin (OC). Probio-M8 improved microbial interactions (e.g., among *Bifidobacterium*, *Eubacterium*, *Cyanobacterium*, and *Ruminococcus*) within the GM of PMOP, specifically enhancing butyrate synthesis while diminishing the prevalence of *Ruminococcus*, which has been inversely connected with OP (71). In a study investigating the relationship between altitude, bone density, and GM among diverse ethnic groups in China, Haojiang Zuo et al. (112) found that altitude was negatively correlated with BMD. Individuals at lower altitudes demonstrated increased gut microbial richness and evenness, as well as markedly elevated total concentrations of SCFAs—including acetate, butyrate, valerate, isovalerate, and isobutyrate—relative to those at high elevations. *Catenibacterium* had a positive correlation with altitude but a negative correlation with bone density. The findings indicate that high-altitude exposure may decrease BMD and elevate OP risk in adult populations, whereas modulation of the GM could act as an effective strategy to alleviate this condition.

SCFAs exert a substantial regulatory influence on both body weight and bone health (113). Plasma butyrate concentrations are inversely associated with obesity (114). Although Sanna S et al. (115) discovered nine genetic polymorphisms linked to butyrate production, no research has investigated the impact of SCFA-related genetic variations on changes in bone density following weight reduction. Tao Zhou et al. (116) discovered that changes in BMD throughout dietary weight-loss treatments correlated with dietary fiber consumption and sex—a relationship presumably influenced by genetically determined butyrate concentrations. This indicates that individuals with higher butyrate production may more effectively preserve bone health while increasing dietary fiber during weight loss. SCFAs demonstrate sex-specific effects on bone health (116). Reduced estrogen concentrations drive enhanced bone resorption and suppress bone formation, ultimately leading to progressive bone loss (117). Butyrate can increase estradiol secretion and assist in sustaining circulating estrogen levels (118), which may be conducive to bone health. In one investigation examining GM-induced insulin-like growth factor-1 (IGF-1) production and bone formation, Jing Yan et al. (116) observed that the administration of a combination of antibiotics (0.2 mg/mL gentamicin, 0.15 mg/mL ciprofloxacin, 2 mg/mL streptomycin, 1 mg/mL bacitracin) to specific pathogen-free (SPF) mice diminished serum IGF-1 levels and reduced bone formation. However, supplementation with normal concentrations of SCFAs (67.5 mM acetate, 40 mM butyrate, 25.9 mM propionate) mitigated this inhibitory effect (119). Tyagi AM et al. (106)demonstrated that oral administration of *LGG* increased the prevalence of butyrate-producing *Clostridium* in the gastrointestinal tract, elevated butyrate concentrations in the intestine and blood, and enhanced regulatory T cell (Treg) populations in both the gut and bone marrow. This induced CD8+ T cells to secrete Wnt10b, hence enhancing bone formation in young mice through the stimulation of Wnt signaling in osteoblasts. Reducing *LGG* or butyrate diminished Treg counts and eliminated the pro-osteogenic effects. These data indicate that *LGG* and butyrate supplementation may potentially be used to prevent or treat OP. However, extensive clinical trials are needed for validation. Mesenchymal stem cells (MSCs) can develop into osteogenic or adipogenic lineages. Research by Chen et al. (120) demonstrated that sodium butyrate stimulates osteogenic differentiation in MSCs via enhancing *Runx2* and its downstream target genes, while also influencing MSC differentiation through extracellular signal-regulated kinase (ERK) activation.

It is important to recognize that most fermenting bacteria target the colon (121), and the effects of SCFA supplements on bone metabolism may vary based on the route of administration (122), contrasting with the actions of microbially produced by microorganisms. Furthermore, SCFAs may exhibit distinct functions across several tissues or even among cell types within the same tissue (123). Further research is needed to clarify the underlying mechanisms by which these factors regulate host physiological processes and pathological conditions. In the context of OP, SCFAs probably affect bone remodeling via several organs (124). Examining the spatiotemporal concentration and functional roles of gut microbial metabolites will help clarify their processes in host bone health.

### 5.2 Lipid metabolism

Dietary lipids account for roughly 35% of the daily energy consumption in humans (125). Owing to their insolubility in water, the digestive tract conveys lipids as lipoproteins, establishing a complex system for lipid digestion and absorption (126). Lipoprotein particles are produced in the liver and intestine, consisting of lipids (such as phospholipids, cholesterol, and triglycerides) and apolipoproteins (127). The gut serves as the principal site for fat breakdown and absorption, but also harbors a diverse microbiota. GM can modulate the content, digestion, and absorption of dietary lipids, potentially altering the formation of intestinal lipoproteins (128). In 2004, Jeffrey Gordon and colleagues initially documented a notable disparity in body fat content between germ-free mice and conventionally raised mice with normal GM, with the latter exhibiting 40% greater body fat (129). Subsequently, an increasing body of GM in lipid metabolic health.

In obese mice, the prevalence of *Bacteroidetes* decreases by 50%, although *Firmicutes* increase correspondingly (130). Similarly, obese individuals exhibit an altered ratio of *Bacteroidetes* to *Firmicutes*, which can be ameliorated by a low-calorie diet (131). HFD-induced changes in GM lead to a heightened prevalence of lipopolysaccharide (LPS)–producing bacteria, correlating with elevated risks of hyperglycemia and hyperinsulinemia (132). One hundred forty microbial-derived sphingolipids produced from microbes can influence the lipid metabolism of the host (133). The GM of healthy individuals can confer protection against metabolic disorders. Fecal microbiota transplantation (FMT) from healthy donors to individuals diagnosed with metabolic syndrome, delivered via duodenal infusion, has been shown to alter the gut microbial profile of recipients and enhance insulin sensitivity (134). Research involving animals suggests that specific bacterial species, such as *Akkermansia muciniphila* (*A. muciniphila*), are beneficial against diet-induced obesity, fasting hyperglycemia, and adipose tissue dysfunction (135). Obesity, hyperglycemia, and dyslipidemia are established risk factors for OP, and persons who are obese and diabetic frequently present with concomitant OP (136). Interestingly, an increased fat mass was once considered to confer protection for bone health (137, 138). Possible mechanisms by which GMs may improve bone metabolism by the modulation of glucose and lipid metabolism include (139): GMs influence host lipid metabolism by altering bile acid composition and bile acid receptor signaling (140); GM regulates bile acid homeostasis, influencing several host pathophysiological processes (141). Alterations in GM influence both bile acid composition and also bile acid receptor signaling, significantly impacting lipid metabolism (142).GM modulates lipid metabolism by influencing levels of microbially produced SCFAs (143); GM influences lipid metabolism by affecting the amount of intestinal barrier function (144).

Research conducted by Xuemei Yuan and colleagues revealed that rats with prednisolone-induced OP displayed markedly increased plasma concentrations of lysophosphatidylcholine (LPC) and phosphatidylcholine (PC), linked to gut microbial metabolism. Both LPC and PC are classified within the glycerophospholipid group, and their increase may be ascribed to heightened oxidative stress arising from an excess of ROS (145). Increased oxidative stress inhibits the differentiation of osteoblasts and induces their apoptosis (146). Under typical circumstances, the body sustains a balance between oxidation and antioxidation, preventing ROS overaccumulation and subsequent disruption of bone metabolism. However, prednisolone not only directly interferes with bone remodeling by affecting osteoblasts and osteoclasts but also precipitates disturbances in lipid metabolism (147). *Prevotella* and *Bacteroides* are intricately associated with obesity: *Prevotella* predominantly metabolizes proteins and fats (148), while *Bacteroides* processes carbs and fiber. Specific members of the Lachnospiraceae family, such as *Blautia*, *Dorea*, and *Ruminococcus*, similarly impact body mass index and weight gain (149). In contrast, *Oscillospira* is negatively correlated with obesity-related genes (150). Danjun Guo et al. (151) reported elevated levels of *Veillonellaceae* and *Prevotellaceae* in the gastrointestinal tract of OVX osteoporotic rats. Supplementation with VSEE (Val-Ser-Glu-Glu)—a bioactive peptide derived from duck egg white—enhanced BMD and alleviated dyslipidemia in experimental models. It markedly diminished the concentrations of *Veillonellaceae*, *Prevotellaceae*, *Fusobacterium*, *Ruminococcus*, and *Roseburia*, while increasing *Bacteroides*. Further mechanistic investigations demonstrated that VSEE enhances the amounts of Wnt3a and LRP-5 proteins, thus stabilizing β-catenin. As a classic ligand of the Wnt/β-catenin signaling pathway, Wnt3a suppresses adipogenic differentiation of MSCs via the downregulation of peroxisome proliferator-activated receptor γ (PPARγ) and CCAAT/enhancer-binding protein α (C/EBPα) (152), thereby playing a crucial role in protecting against bone loss and regulating lipid metabolism in OVX rats. OVX osteoporotic rats frequently have GM dysbiosis-induced lipid metabolic disorders (153), which might impair bone marrow microcirculation and lead to bone loss (154, 155). Research conducted by Hui-Hui Xiao and colleagues (156) demonstrated that OVX rats exhibited dyslipidemia, abnormal bile acid metabolism, and insulin resistance, which were correlated with an elevated *Firmicutes*/*Bacteroidetes* (F/B) ratio — a well-recognized biomarker for steroid deficiency-induced metabolic and skeletal disorders. The intervention with a lignan-rich fraction from S. williamsii restored microbial composition, increased the prevalence of *Actinobacteria*, diminished dyslipidemia, improved liver function, suppressed glucose intolerance and insulin resistance, lowered systemic inflammation, and fortified intestinal barrier integrity, ultimately producing anti-osteoporotic effects. These outcomes offer novel insights into innovative strategies for the prevention and treatment of OP through the targeting of GM-regulated lipid metabolic pathways.

### 5.3 Trimethylamine N-Oxide

Foods including meat and eggs—especially fish and other seafood—are abundant in carnitine and choline (157). These chemicals are degraded by gut microbial enzymes (choline-TMA lyases) into trimethylamine (TMA), which subsequently enters the portal circulation and is oxidized in the liver by flavin-containing monooxygenases (FMOs) to produce TMAO (158). Choline-TMA lyases exist in three major bacterial phyla: *Firmicutes*, *Proteobacteria*, and *Actinobacteria* (159). HFD may be an additional factor influencing circulating TMAO levels (160). It diminishes mitochondrial function in colonic epithelial cells, elevating intestinal oxygen and nitrate concentrations. This promotes the growth of *Escherichia coli* and enhances choline degradation by upregulating the *cutC* gene, which is implicated in the transformation of choline to TMA, hence increasing TMAO levels (161). Circulating TMAO has been identified as a risk factor for various chronic conditions, including kidney disease, type 2 diabetes (T2DM), musculoskeletal disorders, and cancer (162).

HFDs modify the polyadenylation of γ-butyrobetaine hydroxylase (*Bbox1*) mRNA (163), facilitating the production of L-carnitine from γ-butyrobetaine. L-carnitine may influence bone health by decreasing bone turnover and promoting osteoblast activity (164, 165). Nonetheless, high-fat diets may negatively impact skeletal health by promoting the gut microbial transformation of dietary L-carnitine into TMAO (166), so disrupting L-carnitine’s advantageous effects and eventually influencing BMD. A study by Tao Zhou et al. (167) suggested that TMAO might confer a preventive benefit against BMD loss during weight reduction. Decreases in plasma TMAO were associated with BMD loss, irrespective of changes in body weight, diabetes condition, or blood glucose levels. The association between alterations in plasma L-carnitine levels and BMD was further modulated by dietary fat intake. Conversely, Yakun Liu et al. (168) reported markedly raised blood TMAO levels in PMW with hip fractures, suggesting that heightened TMAO may be associated with an increased risk of OP and fracture in this population. Li L et al. (169) demonstrated that feeding Institute of Cancer Research (ICR) mice D-galactose/sodium nitrite for 90 days promoted aging-related OP and cognitive decline. This phenotype was defined by increased oxidative stress, presumably influenced by altered GM (e.g., reduced *Bifidobacterium*, elevated F/B ratio) and raised concentrations of FMO3 and TMAO. Increased oxidative stress further accelerated aging by raising serum TNF-α levels, reducing Sirtuin 6 (Sirt6) expression in long bones, and promoting NF-κB acetylation and *cathepsin K* overexpression and activation.

Whether TMAO is a causative factor in the development and advancement of OP or merely a biomarker of underlying pathology remains unclear. Blood and urine levels of TMAO are influenced by GM, liver and kidney function, diet, aging, FMO3 activity, and/or genotype (170). TMAO concentrations correlate positively with various pathological conditions, including cardiovascular disease, diabetes, cancer, autoimmune disorders, bone metabolic diseases, polycystic ovary syndrome, and autism, while showing a negative correlation with conditions such as dementia and IBD (171). Nevertheless, research findings across these diseases are inconsistent, highlighting the complexity of host-microbiota interactions (172). The role of GM-derived TMAO in OP necessitates further exploration.

### 5.4 Genetic information

Research demonstrates that the host’s inherent genetic traits significantly influence the abundance of the GM and susceptibility to pathogen colonization (173, 174). Another factor affecting the species diversity of the host’s GM may be sex (175). Scientists frequently describe the GM as humanity’s “second genome” (176). Metagenomic sequencing has revealed almost 170 million protein sequences, exceeding 200,000 non-redundant genomes (177), more than 140,000 phage species, and 4,644 prokaryotic species from over 20,000 human gut microbial samples globally (178). The gut microbial population harbors a vast gene pool that influences the diversity of messenger RNA (mRNA), DNA methylation, and non-coding RNAs (such as miRNAs, lncRNAs, and circRNAs) within the host (177, 178).

Genetic background influences baseline bone mass and the distinct distribution of intestinal antigen-presenting cells (APCs) with various roles. Gut APCs, particularly dendritic cells (DCs), exhibit pathogenic antigens derived from the GM and stimulate CD4+ T cells to secrete pro-inflammatory cytokines, including tumor necrosis factor-alpha (TNF-α). These cytokines promote osteoclastogenesis and precipitate bone loss (179). Xin Xu et al. investigated the association between prevalent variations in R-spondin/Wnt signaling genes, GM makeup, and OP risk in an older Han Chinese cohort. They found that genetic variants in LGR6 (rs10920362) and LGR5 (rs11178860) were strongly associated with OP risk. Various microbial species exhibited correlations with BMD and T-scores across multiple skeletal locations. The correlation between rs10920362 and BMD-related microbiota remained significant. In comparison to the CC genotype, the CT/TT genotype of rs10920362 was associated with reduced presence of *Actinobacteria* and *Bifidobacteriaceae*, suggesting that variations in *LGR6* may influence OP pathogenesis through modulation of the GM (180). Genetic polymorphisms and changes can alter GM composition and affect OP risk. Research by Li S et al. (181) showed that polymorphisms in genes of the R-spondin/Wnt signaling network are highly correlated with GM composition. Genetic differences within this network may affect the risk of OP by modifying the GM. Consequently, preventing and treating primary OP necessitates an evaluation of both genetic predisposition and the GM. The identification of connections between host genes and the GM provides novel perspectives for addressing refractory OP and supports the development of future microbiota-focused treatment approaches.

The GM can promote genetic changes in IECs (182), though the exact pathways are not fully comprehended. Specific microbial communities seem to facilitate epigenetic and genetic alterations. Recent investigations utilizing Caco-2 cell models have shown that *Bifidobacterium lactis* upregulates cyclooxygenase-1 (Cox-1) and downregulates Cox-2 gene expression (183). GM organisms may reduce DNA damage, protect the intestinal barrier, enhance mineral absorption, and contribute to the prevention of OP (184). A key method via which microbial-derived SCFAs influence bone metabolism involves the direct modulation of proteins related to calcium absorption. *In vitro* experiments and *in vivo* studies employing animal models have demonstrated that SCFA supplementation elevates the transcriptional expression of TRPV6 and calbindin-D9k in both human colonic epithelial (Caco-2) cells and the colonic mucosa of rats (108, 185). The TRPV6 gene possesses components that react favorably to SCFAs, and the response of calbindin-D9k to SCFAs is contingent upon both time- and concentration-dependent factors. Prebiotic diets markedly enhance calbindin-D9k expression in the colorectum, regardless of dietary calcium intake or serum 1,25-(OH)_2_D levels. This process involves the transcription factors VDR and CDX-2. Butyrate, an SCFA derived from prebiotic fermentation, enhances VDR, activates the CDX-2 promoter, and stimulates CDX-2 mRNA expression (186).

GM may potentially affect OP development by modulating host miRNAs, which are non-coding RNA molecules that contribute to post-transcriptional gene regulation. Multiple miRNAs, such as miR-33-5p (187), miR-194 (188), and miR-433-3p (189), influence genes associated with osteoblast development in OP. Although not completely clarified, GM dysbiosis may alter host miRNA profiles, potentially inhibiting osteoblast development and presenting a challenge for OP treatment (190). Furthermore, the host’s genetic composition influences the GM, and the development and progression of postmenopausal OP (PMO) are contingent upon the interactions between GM and host genetics. Sex is a significant factor influencing microbial diversity (107). Genetic background substantially influences the rate of bone loss in PMO, and bone resorption during PMO is strongly heritable (191). Research demonstrates that estrogen deficiency-induced bone loss due to estrogen shortage significantly differs among mouse strains, suggesting that genetic factors regulate PMO bone loss via various pathways (53).

Genetic background influences bone loss in PMO by affecting the GM, establishing baseline bone mass, regulating the distribution of antigen-presenting cells (APCs), and influencing the development and activity of the host immune system. An expanding body of literature emphasizes the essential role of GM in modulating bone mass throughout development and disease states, typically alongside modifying factors like dietary intake, genetic background, lifestyle habits, and pharmacological treatments (192).

## 6 Impact of GM on vitamins and minerals

**Figure 3:**
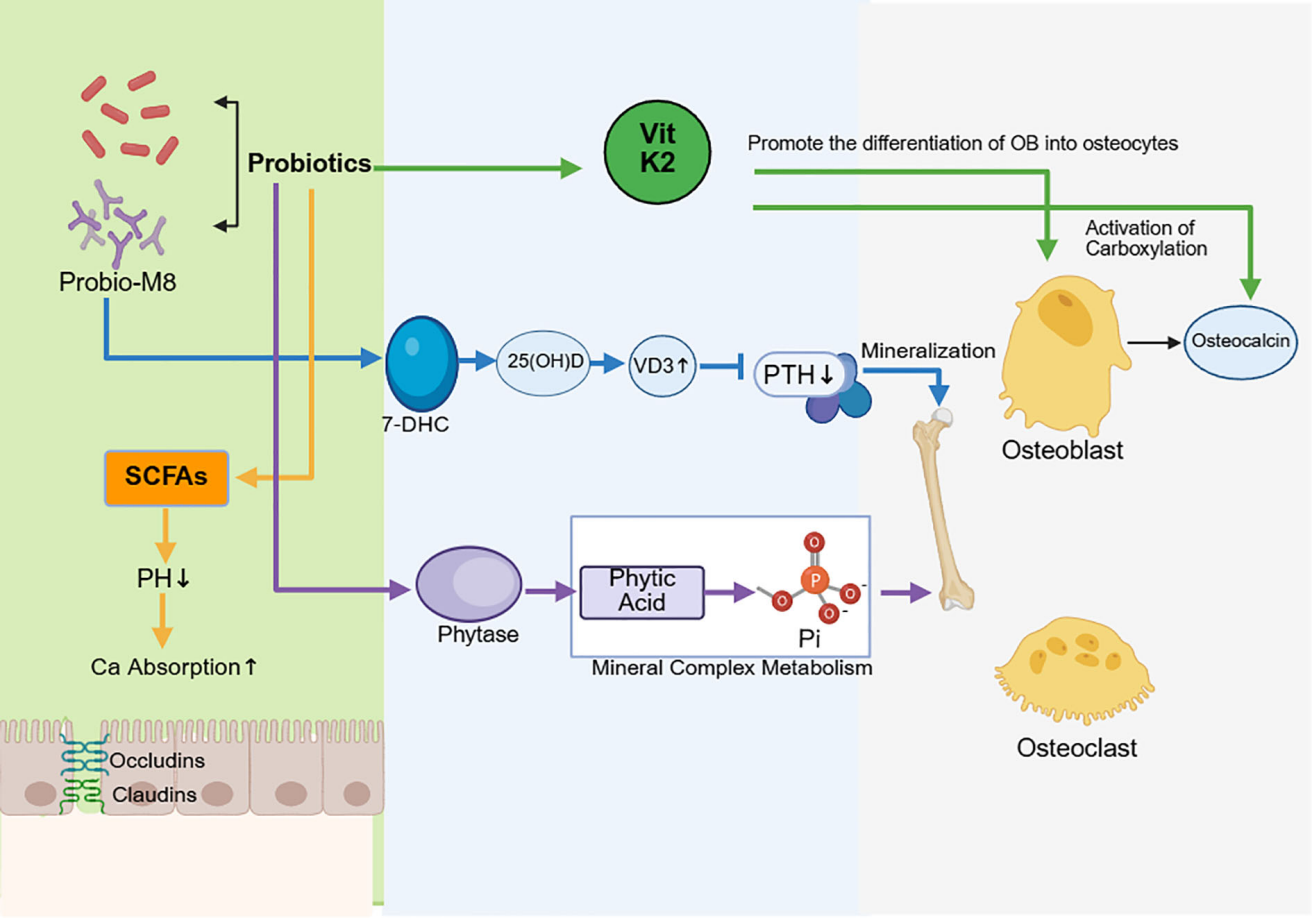
How gut microbiota affects bone metabolism. Created in Biorender.com.

The GM functions as both a “metabolic organ” and a “mineral-processing bioreactor,” regulating the bioavailability and skeletal distribution of vitamins and minerals (**Figure 3**). The GM serves as a major source of several vitamins, including B vitamins and vitamin K (193). Furthermore, it can alter intestinal VitD metabolism, and probiotic supplementation has been shown to affect circulatory VitD levels (37). These vitamins could exert direct and/or indirect impacts on skeletal biology and the bone extracellular matrix. Conversely, dietary calcium may demonstrate prebiotic-like effects by altering the composition and structure of the GM (194). For instance, vitamin K is essential for the functionality of numerous proteins, including osteocalcin—the predominant non-collagenous protein in the bone matrix. The lack of matrix-associated osteocalcin enhances bone brittleness and elevates susceptibility to fractures (195).

### 6.1 Vitamin absorption

A mutually beneficial interaction between the host intestinal tract and GM is critical for breaking down nutrients that cannot be directly assimilated. Osteocalcin is a protein that binds calcium to the bone mineral matrix. Vitamin K_2_, which enhances bone health through osteocalcin, is partially derived from the GM. Vitamin K_2_ is required for osteocalcin function and may stimulate the transformation of osteoblasts into osteocytes. Vitamin K is associated with changes in the composition and structure of organic or mineral components of bone, influencing bone formation by limiting osteoclastogenesis and mediating the carboxylation of osteocalcin. Moreover, research indicates that reduced vitamin K_2_ levels resulting from antibiotic-induced microbiota depletion are associated with decreased osteocalcin and bone strength in mice (196). According to a study in healthy children and adults, children exhibit significantly elevated levels of undercarboxylated osteocalcin—generally 8–10 times more than those found in adults—implying a reduced vitamin K status and greater vitamin K demand in the pediatric population (197).

Calcium and VitD play crucial roles in bone metabolism, including the remodeling of mature bone and the formation of new bone (198), and are equally important in maintaining GM equilibrium. A correlation occurs between VitD and the GM; low VitD levels may elevate intestinal permeability, leading to a persistent low-grade inflammatory state (199). Calcium and VitD are conveyed by paracellular and transcellular mechanisms across the small intestinal barrier, and the GM facilitates the absorption of these vital nutrients. Evidence indicates that the GM markedly elevates circulatory 25-hydroxyvitamin VitD levels by promoting the production of lactic acid and 7-dehydrocholesterol, therefore aiding skeletal calcium absorption (200). Under classical VitD deficiency, serum 25(OH)D levels are below 30ng/mL (75nmol/L). However, the above threshold must now be interpreted carefully. The newly reorganized 2024 Endocrine Society Clinical Practice Guideline suggests that advocates for the general population to maintain 25(OH)D levels above 30 ng/mL is no longer recommended (201). Rather, the present data would support a lower threshold of 20ng/mL (50nmol/L), which is typically optimal for the majority to maintain bone health. Among various factors, the guideline dismisses routine testing to achieve certain levels that are specific to each healthy individual and must be personalized rather than standardized.

Feiyan Zhao et al. (78) demonstrated that *Bifidobacterium lactis* Probio-M8 increased serum vitamin D_3_ levels and decreased PTH levels in subjects. Elevated serum VitD promotes calcium deposition in bone; thus, increased vitamin D_3_ and decreased calcium in the Probio-M8 group suggest greater bone formation activity. Additionally, probiotics may increase serum vitamin D_3_ levels and assist in the prevention of bone loss. Deficiency in active VitD (1,25(OH)_2_D) or VDR leads to gut dysbiosis in mice. In models of IBD, VitD suppresses Th1/Th17 cell populations and induces Treg cells, further underscoring the important roles of GM, calcium absorption, and VitD in regulating bone metabolism (202).

### 6.2 Mineral absorption

In addition to vitamins, the GM significantly affects mineral absorption. Bone is a vital organ composed primarily of minerals arranged around a protein matrix. The key minerals within bone are calcium and phosphorus, which impart “hardness,” while a robust collagen network confers “flexibility.” The GM promotes mineral absorption in the intestine by stimulating the proliferation of enterocytes and colonocytes, therefore facilitating microbial homeostasis (203). Beneficial bacterial communities in the GM digest dietary fiber to produce SCFAs. SCFAs decrease the local pH within the gastrointestinal tract, inhibiting the formation of insoluble calcium-phosphorus complexes and consequently enhancing intestinal calcium uptake (204). They also enhance the solubility of inorganic salts, thereby facilitating their uptake across the intestinal mucosa (205). Additionally, GM-synthesized phytase metabolizes phytic acid—a mineral-chelating compound—into inorganic phosphate and inositol phosphate derivatives (206). Inorganic phosphate is essential for osteoblast-mediated mineral deposition and bone homeostasis (207). Butyrate, a type of SCFA, serves as an energy source for IECs, improves villus structure, enhances intestinal elasticity, and increases the absorptive surface area, hence promoting calcium absorption. Species within *Lactobacillus* and *Bifidobacterium* contribute to efficient uptake and utilization of trace minerals, including calcium, phosphorus, and magnesium, therefore boosting the production of calcium-binding proteins and enhancing bone mineralization (208).

## 7 GM influences bone metabolism via systemic immunity and inflammation

**Figure 4:**
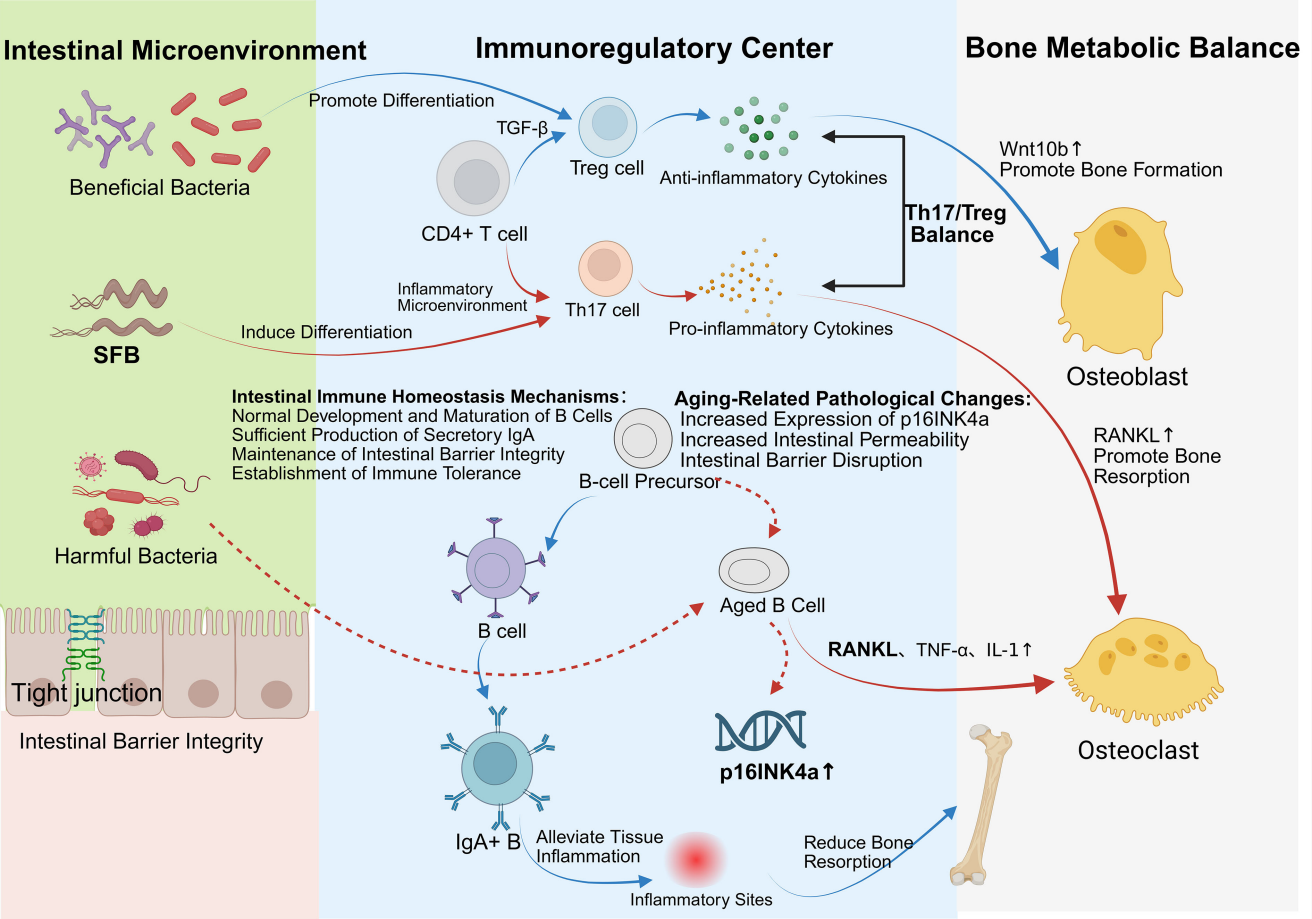
Roadmap for GM-targeted therapies in OP. Created in Biorender.com.

The immune system’s principal job is to differentiate between bacteria typically found in the host environment and invading pathogens. The GM, obtained from the environment, can initiate immune responses both locally in the colon and systemically when its composition alters (209). The GM has demonstrated interaction with immune cells and modulation of key signaling pathways pertinent to both innate and adaptive immunity (52). It may additionally influence bone metabolism through immune system modulation—via the secretion of microbial metabolites that interact with immune cells (e.g., T cells, B cells), activate the intestinal mucosal immune response, and induce the production of pro- or anti-inflammatory mediators and cytokines involved in the regulation of bone remodeling (210). The schematic representation of these multi-step immune–bone interactions is illustrated in **Figure 4**. The GM is a crucial regulator of osseous metabolism. Under conditions of dysbiosis, it may contribute to bone loss in OP by modifying immune function and increasing the production of bone-metabolism-related cytokines. Germ-free mice have increased trabecular bone mass and mineral density relative to traditionally grown mice, with no alterations in bone production. Simultaneously, these “bacteria-free” animals have an underdeveloped intestinal mucosal immune system and systemic immunological modifications, including a marked decrease in T helper cells in the spleen. This suggests that the GM facilitates the growth of the systemic immune system (210), indicating a connection between microbial colonization, immune cell development, and OP pathogenesis. Pamela Schnup et al. found that transplanting a complex microbiota into germ-free mice alleviated the depletion of bone marrow progenitors and restored monocyte proliferation (211). As noted by Iddrisu Ibrahim et al. (212), “The GM is crucial in regulating both innate and acquired immunity, while simultaneously generating immune intermediates that are indispensable for the development of pro- and anti-inflammatory responses.”

### 7.1 Modulation of T cell responses and differentiation

The GM is intricately associated with the equilibrium between T helper 17 (Th17) cells and Tregs, suggesting that intestinal bacteria may provoke a disruption in Th17/Treg immunity (213). Tregs and Th17 cells are two functionally antagonistic lymphocyte subsets originating from the same precursor (214), with their development controlled by TGF-β (215). Both are crucial in sustaining bone homeostasis, especially in modulating osteoclast differentiation (216). GM dysbiosis can enhance the formation and development of Th17 cells, a subset of CD4+ T cells. Such cells secrete IL-1, IL-17a, IL-6, small amounts of interferon-γ (IFN-γ), and tumor necrosis factor (TNF), which facilitate the liberation of RANKL, strengthen osteoclastic action, and exacerbate bone loss. Tregs, conversely, enhance osteogenic differentiation by inducing bone marrow CD8+ T lymphocytes to produce Wnt10b, a ligand of the osteogenic Wnt signaling pathway (217). Also, Tregs suppress osteoclastogenesis by inhibiting the expression of Traf6 and Nfatc1, as well as RANKL-stimulated NF-κB signaling, thereby promoting bone formation and regeneration (218).

The Th17/Treg balance can be restored by specific gut microorganisms such as Lactobacillus acidophilus, which promotes the secretion of anti-inflammatory cytokines, such as TGF-β and IL-10. This inhibits osteoclast proliferation, differentiation, and bone resorption, raises bone mass, and may contribute to the prevention of OP (219). Studies have revealed that one effect of low estrogen concentrations in PMW is GM dysbiosis, which leads to elevated levels of osteoclastogenic cells (Th17 cells, CD4+ T cells) and the pro-osteoclastogenic cytokine TNF-α. This is one of the pathways of GM-mediated bone resorption in PMO. Intriguingly, intestinal colonization of germ-free mice with bacteria such as Clostridium increases latent TGF-β, promotes Treg cell development, and enhances bone mass and immune regulation. Conversely, an unbalanced gut microbial population can prevent the activation of Th17 cells at the expense of Th1, Th2, and Treg cell differentiation.

This promotes osteoclast differentiation and proliferation, accelerating bone loss. Th17 cells are regarded as primary effector cells in the etiology of OP. Comparing germ-free mice to conventionally grown (CONV-R) mice, it has been shown that the former had increased bone mass due to their changed immunological state, which includes lower pro-inflammatory cytokines, decreased CD4+ T cells, and fewer osteoclasts/precursors in the bone marrow (220). *Segmented filamentous bacteria* (*SFB*) in the mouse gut promote the production of IL-17 and IFN-γ and play key roles in both osteoclast and osteoblast formation (221–223), indicating that the GM modulates bone metabolism by altering the host immune status. Remarkably, mice given antibiotics also exhibit better bone mechanical qualities and greater bone mechanical properties. These disparate results might be explained by variables including age, sex, and antibiotic treatment schedules, all of which change the GM and hence affect immune function.

### 7.2 Regulation of B cell function

The intestinal environment is where early B cell growth takes place, and bone marrow immune tolerance mechanisms help make self-reactive B cells tolerant, suggesting that the gastrointestinal tract plays a role in maintaining self-tolerance. Intestinal mucosal homeostasis and the maintenance of regulatory B cell activity depend on ongoing stimulation by gut bacteria (224). Intestinal bacteria have an impact on B cell development, maturation, and activation (225, 226). The GM shapes B cell subpopulations and receptor repertoires. While microbial colonization does not influence the quantity of B cells at different developmental stages in the bone marrow and spleen, it may facilitate B cell maturation in peripheral immune organs, augment the proportion of mature B cells, and enhance the diversity of the B cell receptor (BCR) repertoire. The microbiome also regulates the immune response of B cells. During the study of inflammatory diseases, intestinal IgA B cells are an important source of lymphocytes. Notably, IgA is needed to maintain the GM and intestinal immune homeostasis (227). Such cells can migrate to inflamed tissues and perform regulatory roles, which has a calming effect on inflammatory reactions, indicating crosstalk between GM and B cells in other tissues and also conferring long-term anti-inflammatory benefits (228). Microbial metabolites may also control inflammation in immune-mediated diseases at the molecular level, acting as inherent epigenetic regulators of B cells (229).

The GM maintains the intestinal mucus barrier, stimulates B cells to secrete secretory IgA, and releases various substances necessary for the host’s growth, and inhibits pathogens (230). Probiotics (Bifidobacterium and Lactobacillus) also augment the lymphoid tissues of Peyer’s patches and enhance B-cell function (231). Changes in the GM caused by age, pathogenic invasion, diet, or disease can induce B-cell senescence and consequently weaken the mucus barrier. Bacterial metabolites may subsequently permeate the gut lining and stimulate osteoclasts or inhibit osteoblasts via several routes (232). Research conducted by Shimpei Kawamoto et al. (233) demonstrated an elevated quantity of senescent cells in the ileum of elderly mice. These cells enhance the expression of senescence-induced genes such as *P16ink4a* in B cells, hence expediting systemic aging. The aging process is linked to diminished synthesis and variety of IgA, along with compromised IgA binding specificity to gut bacteria, which disrupts the equilibrium between osteogenesis and osteoclastogenesis and potentially contributes to age-related OP.

Interference with gut ecological balance increases intestinal barrier permeability, allowing bacterial translocation. Translocated microorganisms and their products can activate the immune system. Activated B cells can migrate to bone tissue and directly promote osteoclast formation by releasing osteoclastogenic factors: RANKL, TNF, and IL-1, thereby accelerating bone resorption (234). Increased production of activated B cells can increase TNF-α in the bone marrow, resulting in the differentiation of marrow stem cells into osteoclasts. This disrupts the balance between bone resorption and formation, leading to increased bone resorption and reduced bone density. Another way the GM can impact B cell formation is by regulating the production of osteoprotegerin (OPG), which inhibits bone resorption by osteoclasts (235).

### 7.3 Innate immune sensing and bidirectional crosstalk

Not only does it modulate the T and B lymphocytes, but the innate immunity sensing of gut metabolites by the immune system is also fundamental to bone homeostasis. The gut microbiota-derived Pathogen-Associated Molecular Patterns, including LPS and peptidoglycan, are recognized by Pattern Recognition Receptors (PRRs), in particular Toll-like receptors and NOD-like receptors, which are expressed on osteoclasts and osteoblasts (236). For example, when the environmental conditions change, and the intestinal permeability increases (leaky gut), systemic translocation of a part of GM, which is LPS, directly activates the TLR4 receptor. Such action potently triggers the subsequent NF-kB and MAPK signaling cascades that lead to osteoclastogenesis and quickening of inflammation-associated bone resorption (237). Another exciting trend is that osteoimmunology—the recently discovered crosstalk between gut microbiome and the immune system— has a highly reciprocal character, as both sides of the crosstalk have been proven to be highly active. The host’s immune system actively uses PRR-driven signaling to influence the bone marrow niche, while bacteria’s colonization and proprietary metabolic activity are controlled by the intestinal mucosal immunity system (such as B cell-produced secretory IgA). In a given dynamic, this underlying bidirectional feedback mechanism plays the directing role in the systemic skeletal remodeling (238). In pathological situations, such bidirectional immunological conditions can substantially target the immune system. Immune cells that are directly or indirectly interacting with bacteria mainly influence the gut and the gut’s immune status. Balanced interaction between gut immunity and bacteria decides the success or failure of their ecosystem (239). By way of example, Immune-Mediated Inflammatory disease, which includes Inflammatory Bowel Disease (IBD) and other varieties of gut dysbiosis, may lead to increased intestinal permeability, which allows the initiation and progression of osteoporosis. In contrast to the aforementioned procedures, microbiota-activated T cells may serve as an “inflammatory shuttle”, which is capable of linking the gut and bone directly. Molecular patterns associated with microbes (MAMPs), which we can detect circulating in IBD, might activate immune responses as well as T cells, osteocytes, osteoblasts, and osteoclasts located in the bone marrow, which leads to low bone formation and high resorption (240). Through the regulation of host immunity and the production of microbial metabolites, the GM modulates bone metabolism, slows bone resorption, and may inhibit the development of OP. Conversely, gut dysbiosis can induce immunological activation that enhances osteoclast activity and accelerates bone resorption.

OPG, also known as osteoclast-inhibiting factor (OCIF), is a heparin-binding secretory glycoprotein synthesized by osteoblasts and bone marrow stromal cells. The ligand, OPGL, also known as RANKL, TRANCE, or osteoclast differentiation factor (ODF), is a transmembrane protein expressed by osteoblasts and found in several organs (241). OPG suppresses the development of M cells situated between lymphoepithelial follicles in the intestine, hence contributing to the preservation of intestinal immunological equilibrium. OPG deficiency increases the number of M cells in gut-associated lymphoid tissue (GALT), enhances antibody responses to commensal bacteria, alleviates IBD, and reduces inflammation-induced abnormalities in bone metabolism (235).

## 8 GM modulates bone metabolism through endocrine pathways

**Figure 5:**
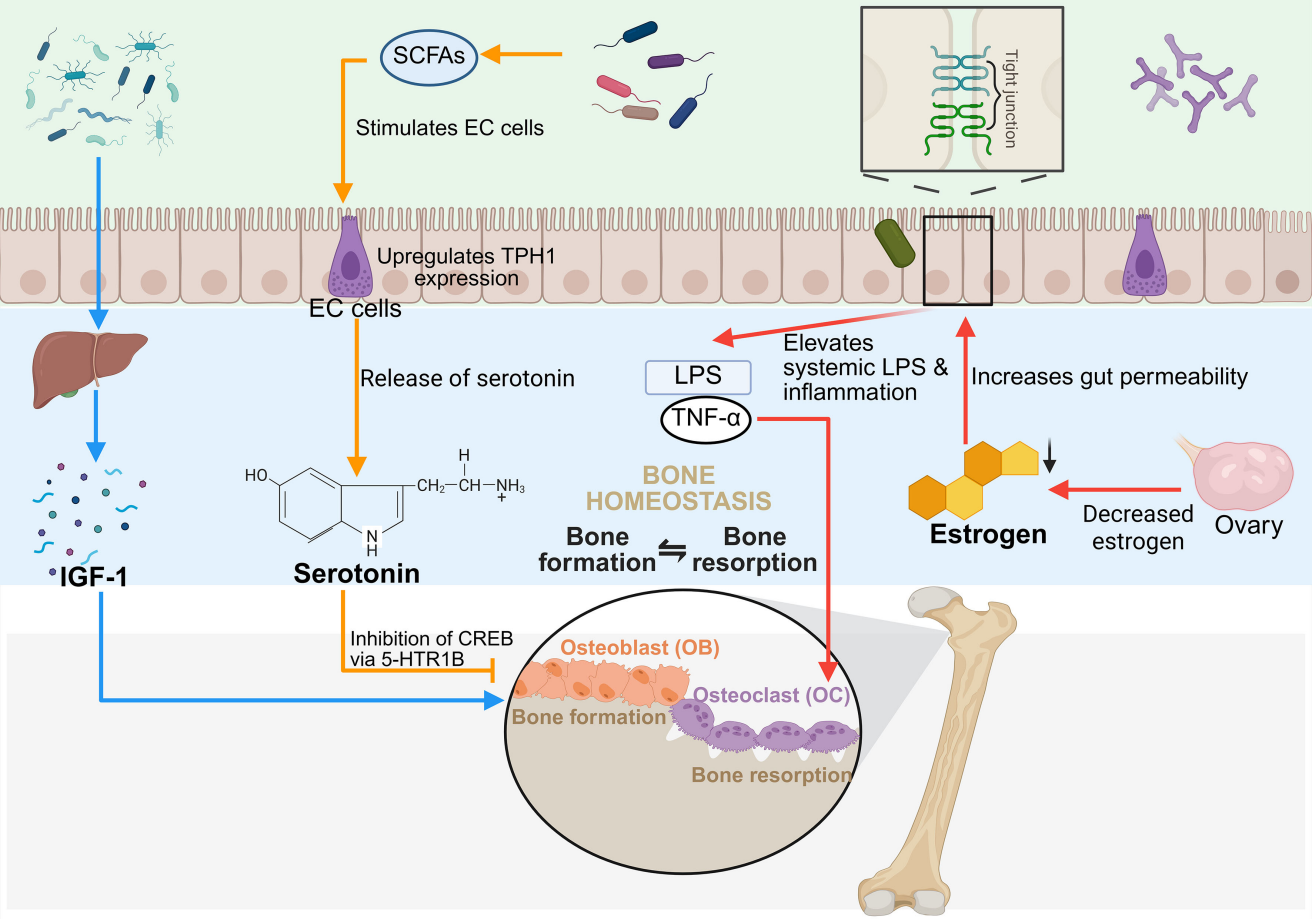
GM regulates bone metabolism via the endocrine system. Created in Biorender.com.

In recent years, the function of GM in modulating bone metabolism through the endocrine system has garnered heightened interest. Numerous studies demonstrate that the GM contributes to bone homeostasis by modulating the release of numerous hormones linked to bone metabolism, such as estrogen (242), IGF-1, 5-HT, PTH, glucagon-like peptide-1 (GLP-1), and leptin (243). These GM–hormone–bone axes are overviewed in **Figure 5**.

### 8.1 Estrogen and age-related inflammaging

The pathogenesis of PMO is deeply intertwined with both age-related senescence and acute estrogen deprivation. The high state in PMW is seen after excessive withdrawal of systemic estrogenic hormones, which deeply affects the physiological process and disrupts the gut milieu and integrity (244). In generic terms, the transcription of necessary tight junction proteins, such as Occludin and ZO-1 fall below given levels due to loss of estrogen signaling in the gut. Hence, estrogen deficiency causes leaky gut, where the microbial products can translocate systemically, provoking the innate immune system via activation of NLRP3 inflammasome, underlying similar events (245).

Thus, a consequential constant leak of microbial entities sustains a persisting low-grade systemic inflammation system referred to as inflammaging. The latter airs such osteoclastogenic factors, especially of TNF-α, IL-1β, and IL-18, which eventually uncouple the bone remodeling and feature as the severity of bone loss in older women. These findings of microbiota-mediated immune facilitations aim at a point that the amelioration of systemic inflammation through the gut-bone axis is a concern for estrogen-associated bone loss patients (246).

The pathomechanisms, which are studied in the scope of clinical trials, are supported by the *in vivo* models. The effects of the GM on the OVX mice’s bone metabolism were evaluated on antibiotic-loading gut flora models. This was noted to raise serum LPS levels and F/B ratio, which is a primary indicator of microbial imbalance that happens with the decrease of estrogen. Antibiotic therapy resulted in augmented osteogenesis and improved characteristics of bone mass and increased overall mechanical resistance. At the same time, the tibial gene expression pattern was restored to an almost average level, observing an increase in periosteal osteogenic activities (247). Hence, the additional findings point out that the presence of GM variations in the menstrual cycle may also be a possible cause of OP, and GM manipulation might help mitigate such negative effects. Besides the 16S rRNA sequencing, GM proportion was determined to be about 60% lower in PMOP compared with PMW, while being reversible by GM intervention. Later on, this was proved in the OVX mouse model that showed GM dysbiosis due to estrogen deficiency brought about by integral bone loss through enhanced gut permeability alongside excessive expression of cytokines, which orchestrated osteoclastogenesis. Thus, this may be interpreted as the direct demonstration of the estrogen, GM, and bone metabolism connection (53).

In clinical settings, researchers investigated 106 PMW with normal bone mass, osteopenia, and OP–using a research method based on 16S rRNA gene sequencing and LC-MS metabolomics –and found that the gut microbiome of patients with PM underwent significant alterations. The research showed a notable decrease in the richness and diversity of the gut microbial population in patients with OP, with structural changes at the phylum and genus levels. The metabolite, on the other hand, which was common in the osteopenia group, was the N-acetylmannosamine and negatively correlated with BMD. In addition to this, the relationship between the relative abundance of bacterial genera, including *Allisonella*, *Klebsiella*, and *Megasphaera*, with the bone turnover marker P1NP and CTX-1, respectively, could therefore imply that these microorganisms were also directly or indirectly engaged in the bone loss process, which was the focus of the study (248). More interestingly, although in the initial stage, the levels of estrogen withdrawal serve as the starting point for enhanced intestinal permeability, several studies have established no significant correlation between the levels of estrogen and the microbial diversity in the established PMO. These findings imply a significant positive effect with age and accelerate inflammation: following the step of initial dysbiosis and inflammation cascade, the altered GM and persistence of inflammation may act synergistically, prolonging the proinflammatory status. Moreover, with relative indifference to subacute decreases of estrogen, the gut dysbiosis stands as a major component in the persistence of bone homeostasis disruption in older PMW, even after the menstrual cycle changes.

### 8.2 Insulin-like growth factor-1.

Many studies show that the GM, IGF-1, and bone metabolism form a complex but important regulatory axis, which is involved in postnatal development and skeletal growth in mammals (249). There is considerable evidence that traditionally bred mice with an intact GM exhibit much higher circulating levels of IGF-1 compared to germ-free (GF) mice. The declining levels of IGF-1 in GF mice are associated with retarded linear growth, reduced body weight, and impaired bone formation–including shorter femur length, lower cortical bone density, and reduced trabecular bone density–all of which indicate that GM plays an important role in optimizing growth. This causal relationship is mediated by the IGF-1 signaling pathway. Functional studies provide tangible data: When GF mice are administered recombinant IGF-1 (to recapitulate the growth-promoting effect of microbial colonization), this has a substantial beneficial effect on body development and femur elongation. Conversely, after blocking the IGF-1 receptor (IGF-1R) in normal mice, the growth advantage conferred by the microbiota is diminished (250). The microbial control of IGF-1 is both dynamic and reversible. Research indicates that colonizing adult GF mice with a conventional microbiota significantly elevates their serum IGF-1 levels, correlating with heightened levels of the bone formation marker P1NP (N-terminal propeptide of type I collagen) and elevated bone formation rates, thereby facilitating both radial and longitudinal bone growth. From another perspective, eliminating GM with broad-spectrum antibiotics, especially vancomycin, which targets Gram-positive bacteria, slows bone growth and lowers blood IGF-1 and P1NP levels (116).

### 8.3 Serotonin (5-Hydroxytryptamine)

5-HT’s distribution and synthesis locations in animals allow it to be classified as either central or peripheral. It is a vital neurotransmitter and paracrine signaling substance that is mostly produced and released by intestinal enterochromaffin (EC) cells (251). It is critical for controlling the growth and neurogenesis of the enteric nervous system, as well as gastrointestinal motility, secretion, inflammatory reactions, sensation, and the development of epithelial cells. Additionally, 5-HT exerts both anti-inflammatory and pro-inflammatory effects under intestinal inflammatory conditions by binding to different receptors, and demonstrates biological functions in systems beyond the gut, such as in autism and depression.

Through its modulation of 5-HT production and secretion, the GM is critical to control bone metabolism. Research indicates that gut microbial metabolites, such as SCFAs and α-tocopherol, can activate various G protein-coupled receptors (e.g., FFAR2, OLF78, GPBAR1) on the surface of EC cells, thereby promoting the expression of tryptophan hydroxylase 1 (TPH1) and the release of 5-HT (252–255). Interestingly, the vast majority of 5-HT is produced by intestinal EC cells. Upon entering systemic circulation and reaching bone tissue, 5-HT attaches to the 5-HTR1B receptor on osteoblasts, which inhibits cAMP response element-binding protein (CREB) activity and cyclin expression, thereby suppressing osteoblast proliferation and differentiation and ultimately hindering bone formation (256). The low-density lipoprotein receptor-related protein 5 (Lrp5) promotes osteogenesis by suppressing TPH1 expression in the gut, therefore diminishing circulating 5-HT levels. The precise regulation mechanisms of the Lrp5–5-HT pathway are still disputed. Furthermore, the connection between the GM and the host not only affects 5-HT production but may also be regulated by feedback from 5-HT levels (251), although this bidirectional regulatory mechanism requires additional exploration.

Furthermore, research revealed that AMP1-1 mitigated OP in tail-suspended rats by influencing the “microbiota-gut-bone” axis, a process intricately linked to the management of GM and reduction of peripheral 5-HT levels (257). AMP1-1 enhanced the intestinal milieu, repaired gut barrier integrity, and reinstated the normal activity of EC cells, therefore diminishing peripheral 5-HT levels. This process was accompanied by substantial changes in GM composition. Moreover, FMT studies validated that the microbiota-modulating properties of AMP1-1 significantly alleviated weightlessness-induced bone loss. These observations provide a novel theoretical foundation for the treatment of OP via targeting the GM–5-HT axis, indicating that suppression of gut-derived 5-HT biosynthesis may serve as a promising anabolic approach for bone health.

## 9 Probiotics and prebiotics

Over the past few years, alongside the progress of gut-bone axis investigations, a mounting body of data has emphasized the notable link between GM and the initiation and advancement of OP. The regulating influence of probiotics and prebiotics on bone metabolism has increasingly emerged as a focal point of investigation. One study systematically demonstrated the feasibility of probiotics and prebiotics regulating OP via the brain-gut-bone axis, particularly emphasizing the central roles of SCFAs, intestinal barrier repair, and immunomodulation, with their efficacy corroborated by both animal experiments and human clinical data (263).

Research in animal models has demonstrated that probiotics can improve osteoporotic characteristics by regulating the GM and immunological equilibrium. For example, *LGG* has protective effects on bone microstructure, bone biomechanics, and related biomarkers (such as CTX-I, PINP, Ca, and RANKL) in an OVX rat model (213). Moreover, *Bifidobacterium animalis* subsp. *Lactis* A6 markedly mitigated muscle and bone degradation in a DSS-induced gut dysfunction model by altering GM composition and promoting butyrate-producing bacteria (264). A separate study indicated that *Bifidobacterium animalis* subsp. *Lactis* HN019 and *Lacticaseibacillus casei* 01 reduced alveolar bone destruction and intestinal permeability while increasing estradiol levels in OVX rats (265). An ongoing double-blind randomized placebo-controlled research is examining the effects of probiotics on BMD and bone metabolism in early PMW, which may validate or contradict the advantages of GM for bone health (266). Although current results from human trials are limited, existing research suggests that probiotics can influence 25-hydroxy VitD levels, calcium intake and absorption, and slightly reduce bone loss in elderly PMW, with effects comparable to calcium and VitD supplementation (35). Additionally, a three-month experiment using *Bifidobacterium animalis* subsp. *Lactis* Probio-M8 (Probio-M8) showed enhancements in bone metabolism in PMOP, while the impact on BMD was not statistically significant (78).

Recent findings underscore the intricacy of probiotic therapies in the management of OP. A randomized clinical experiment examined the impact of *L. reuteri* 6475 on BMD in early PMW. The study indicated that although *L. reuteri* 6475 had demonstrated potential benefits for bone metabolism in animal models and some preliminary human studies, the 24-month double-blind, randomized, placebo-controlled trial showed no significant effects of *L. reuteri* 6475 on total tibial vBMD, lumbar spine, or hip BMD (262). These findings reveal that although probiotics could have some positive impact on bone metabolism in certain specific situations, their effectiveness may vary depending on individual variations, study design, intervention period, and other variables. In addition, 2’-fucosyllactose (2’-FL), a prebiotic, exhibited preventive effects against OP in a naturally aging mouse model by maintaining the diversity and composition of the GM, the gut barrier function, and the innate immune system (267). These results also indicate the potential of using probiotics and prebiotics for the prevention and treatment of OP.

In conclusion, probiotics and prebiotics demonstrate potential beneficial effects on the prevention and treatment of OP through numerous pathways, including manipulation of the GM, enhancement of intestinal barrier function, control of immunological responses, and endocrine influences. Nonetheless, contemporary research is still nascent, necessitating more clinical studies to substantiate their efficacy, resolve concerns about strain selection and dose optimization, and facilitate the clinical implementation of GM-directed therapeutics for OP management (263).

## 10 Nervous system

There exists complicated bidirectional crosstalk among the nervous system, the GM, and skeletal health. The GM communicates extensively with the brain through the vagus nerve (VN) and the autonomic nervous system (ANS) (268, 269). The VN can capture alterations in the gut microbial ecology and transmit these to the central nervous system, which will trigger adaptive or maladaptive responses — the latter potentially leading to gastrointestinal diseases or neurodegenerative diseases (270). The ANS, together with the hypothalamic-pituitary-adrenal axis, represents one large-scale communication system of the host for physiological homeostasis (271) and the regulation of various gastrointestinal functions, including intestinal motility, permeability, and mucosal immunological functions. SCFAs and serotonin are microbe-derived chemical compounds that have the potential to regulate local vagal routes via portal circulation and send signals to the brain through the enteric nervous system (ENS) and vagal afferent routes (268, 269).

The enteric nervous system, as it is occasionally known, controls intestinal function and also influences bone metabolism. The aberrant GM may lead to increased production of amyloid proteins and LPS, increased intestinal permeability, and allow the entry of inflammatory molecules across the blood-brain barrier and thus cause neuroinflammation and neuronal damage (272, 273). The ENS affects bone by secretion of neuropeptides like substance P, which may enhance bone formation (274, 275), and secretion of molecules like vasoactive intestinal peptide, which may suppress osteoclast activity and bone resorption (276). Besides, the ENS exerts an indirect effect on skeletal health via the GM (212). Bone is directly regulated by the neurological system. Osteoblasts and osteoclasts’ functions are regulated by a system of neuropeptides from the central nervous system, which helps maintain the balance between bone formation and bone resorption (277). Sensory nerve fibers and autonomic nerve fibers are involved in bone tissue control (278), but skeletal nerves have the potential to affect intestinal activity indirectly through the ANS and ENS (279, 280). At the same time, bone-derived mediators provide feedback to the brain, influencing its development and activity, which represents significant communication between bone and the brain (277, 281). In short, GM is involved in gut-brain communication via neurological and immunological mechanisms and thus alters bone metabolism and health through the regulation of the enteric nervous system and central nervous system.

## 11 The conceptual framework: frost’s mechanostat theory and the gut-bone axis

The interaction between the GM and homeostasis of bone is more than merely biochemical in nature. Frost’s mechanostat principles offer a convenient physical basis to explain this phenomenon. Bone adapts its mass and structure when mechanical stresses exceed characteristic levels, according to those principles. It has been shown that the GM and its metabolites are systemic factors that can move the mechanosensitive setpoint recently (282, 283).

As the major mechanosensors in bone, osteocytes couple mechanical signals with systemic hormones (284). Depleting gut microbiota greatly reduces the mechanoadaptive response of bone (285). This insensitivity appears to result from specific bacteria, such as Lachnospiraceae and mucin-degrading bacteria (e.g., *A. muciniphila*) that produce L-citrulline and L-arginine. Systemically available L-arginine activates a nitric oxide-calcium positive feedback in osteocytes that heightens the osteogenic response of bone to physiological loading (285, 286). Gastrointestinal endocrine signals traversing this mechanical framework also modulate its function. Physical activity naturally decreases sclerostin and increases serum IGF-1 levels, which promotes bone formation (287). In fact, the *in vivo* studies suggest that an active crosstalk between IGF1 and estrogen is indeed a precondition for cortical bone mechanical strain response (288). Dysbiosis of the gut often occurs in aging or estrogen-deficient situations and results in the disturbance of estrogen metabolism and IGF-1 production. This disruption possibly makes the mechanostat “set-point” shift further upward. The bone, therefore, has to sustain relatively high levels of mechanical strain to avoid further bone loss.

Meanwhile, mechanisms provided by microbial metabolites and the local immune system restore normal mechanosensitivity. Gut microbe-derived SCFAs, such as acetic and isovaleric acid, have a positive association with trabecular bone architecture in early PMW (289). Low-grade systemic inflammation due to dysbiosis has a direct role in the fragility of mechanotransduction. In normal conditions, the loading conditions activate the Piezo1 channel in bone marrow mesenchymal stem cells, which leads to the repression of the Ccl2-Lcn2 inflammatory cascade and a tendency toward osteogenesis instead of adipogenesis (290). The systemic inflammation brought by gut dysbiosis can bypass this mechano-inflammatory immunity. Understanding the gut-bone connection in the context of Frost’s mechanostat theory explains the possible effectiveness of the microbiome modulation approach in restoring the skeletal mechanosensitivity to physical activity.

## 12 GM-targeted therapies: clinical evidence and critical evaluation

Deriving from the existing comprehensive studies on the gut-bone axis, the modulation of the GM presents a promising and increasingly evidence-based strategy for the management of OP in clinical settings. This article extensively outlines the GM’s precise regulation of bone metabolic homeostasis, including intestinal barrier homeostasis control, immune homeostasis, the release of metabolic products (e.g., estrogen, IGF-1, and 5-HT signaling), the endocrine system, and the neural circuitry. Furthermore, growing evidence suggests that the dysbiosis of the gastrointestinal tract may eventually contribute to the impairment of intestinal mucosal integrity, instigate a high-level inflammation response, affect the bile acids’ cross-talk, and disrupt minerals’ absorption rate—thereby, hastening the evolution of OP. An overview of key studies investigating gut microbiota-mediated effects on bone metabolism is provided in **Table 1**.

**Table 1:** Overview of critical research investigating gut microbiota-mediated effects on bone metabolism.

Author	Study population	Main findings	Relevant microbial communities	Mechanisms of Action
Feiyan Zhao (78)	PMO (n =40)	Co-administering Probio-M8 improved the bone metabolism.	*Bifidobacterium lactis* Probio-M8	SCFA↑, vitamin D3↑
Shuai Chen et al. (79)	European ancestry individuals (n = 32,735)	Genetically predicted *Prevotella9* and *Prevotellaceae* levels are associated with increased BMD at the lumbar spine, forearm, and femoral neck.	Genus *Prevotella9*, Family *Prevotellaceae*	Causal relationship between specific GM and BMD; no reverse causality
Manon Lecomte et al. (80)	PMO (n = 100)	8-PN standardized hop extract (HE) increased total body BMD and improved the SF-36 physical functioning score.	Genus *Turicibacter*, Genus *Shigella*	phytoestrogen
Peishun Li et al. (81)	Elderly women with low BMD (n = 20)	One-year supplementation with *Lactobacillus reuteri* ATCC PTA 6475(*L. reuteri* 6475) increased GM gene richness, improved the inflammatory state, and reduced *E. coli* and its biofilm formation.	*L. reuteri* 6475, SCFA-producing bacteria (e.g., *C. acetobutylicum*, *A. fermentans*, *A. muciniphila*, *C. catus*, *R. bicirculans*)	*L. reuteri* 6475 may promote bone formation by modulating GM composition and function, reducing bone loss.

### 12.1 Clinical evidence of probiotics, prebiotics, and synbiotics

Interventions that aim at modulating the gut microbiome, such as targeted probiotics (291), prebiotics (263), postbiotics (292), balanced diets (293), or microbiota transplants (294), are available. Whereas preclinical and clinical research have extensively expounded on these target organ interventions, clinical application and instance-based evidence on clinical translatability are needed. An increased number of randomized controlled trials (RCTs) on the influence of targeted GM therapies. Good-quality clinical data is now available that shows enhanced regulation of bone turnover. Current clinical data, including a high-quality meta-analysis, strongly suggest that targeted probiotics therapy can modulate markers of bone turnover (BTMs): BTM markers are decreased for resorption, such as CTX-I and TRAP-5b, while they remain unchanged for formation, such as P1NP or osteocalcin, in a meta-analysis of 2026 RCTs (n = 1432) (295). Additionally, parallels are drawn between promising therapies of probiotics and prebiotic fiber to produce “synbiotics”: probiotics combined with prebiotic fiber demonstrate improved clinical outcomes. Synbiotic medical foods not only prevent bone loss but also improve gut permeability and decrease cytokines produced by systemic immune cells’ activity; these effects flow in parallel, thereby establishing a clinical outcome link at the individual level among osteopenic women (296). The effects of probiotics and prebiotics on bone metabolism from clinical trials are summarized in **Table 2**.

**Table 2:** Effects of probiotics and prebiotics on bone metabolism.

Author	Study population	Probiotic/Prebiotic type	Main findings	Mechanisms of action
A G Nilsson et al. (258)	Women aged 75-80 with low BMD (n = 90)	*L. reuteri* 6475	After 12 months, *L. reuteri* 6475 significantly reduced the loss of tibia total volumetric bone mineral density (vBMD).	Not specified
Marut Vanitchanont et al. (259)	PMO (n=40)	Multispecies probiotics	After 12 weeks, multispecies probiotics significantly decreased serum CTX.	Preventive effect on bone through antiresorptive action.
Takuou Takimoto et al. (260)	Healthy postmenopausal Japanese women (n = 76)	Bacillus subtilis C-3102	After 24 weeks, C-3102 significantly increased total hip BMD, decreased urinary type I collagen cross-linked N-telopeptide (uNTx), and decreased tartrate-resistant acid phosphatase isoform 5b (TRACP-5b).	Improved BMD by inhibiting bone resorption and modulating GM.
Iskandar Azmy Harahap et al. (261)	PMW (n = 55)	*Lactobacillus acidophilus*	After 12 weeks, L. acidophilus significantly decreased serum calcium levels but had no significant effect on bone metabolism biomarkers or BMD.	Modulated GM and calcium metabolism to affect bone metabolism, but may have adverse effects on glucose metabolism.
Giulia Gregori et al. (262)	Early PMW (n = 239)	*L. reuteri* 6475	After 24 months, *L. reuteri* 6475 had no significant effect on tibia total vBMD, lumbar spine, or hip BMD.	Not specified

### 12.2 Critical appraisal and future interventions

However, despite the described clinical improvements, the current situation in clinical practice has some issues. According to a recent review (297), clinical limitations often include heterogeneity of probiotic strains, large dose variability, and limited follow-up. Importantly, while these results are encouraging, they should be interpreted with caution. Most current clinical studies focus primarily on biochemical markers or BMD as surrogate endpoints. Further large-scale, long-term longitudinal trials are required to confirm whether these microbiota-based interventions directly translate into a reduced risk of clinical fractures—the ultimate gold standard for OP treatment. Furthermore, the profound osteogenic properties of FMT treatment, which appear to entirely remodel the mouse immune-microbiome ecosystem, are not yet applicable for human OP intervention due to safety and regulatory concerns. Consequently, there are problems for their use as therapies that are related to variability of strains, variability of hosts, and the need to standardize procedures.

**Figure 6:**
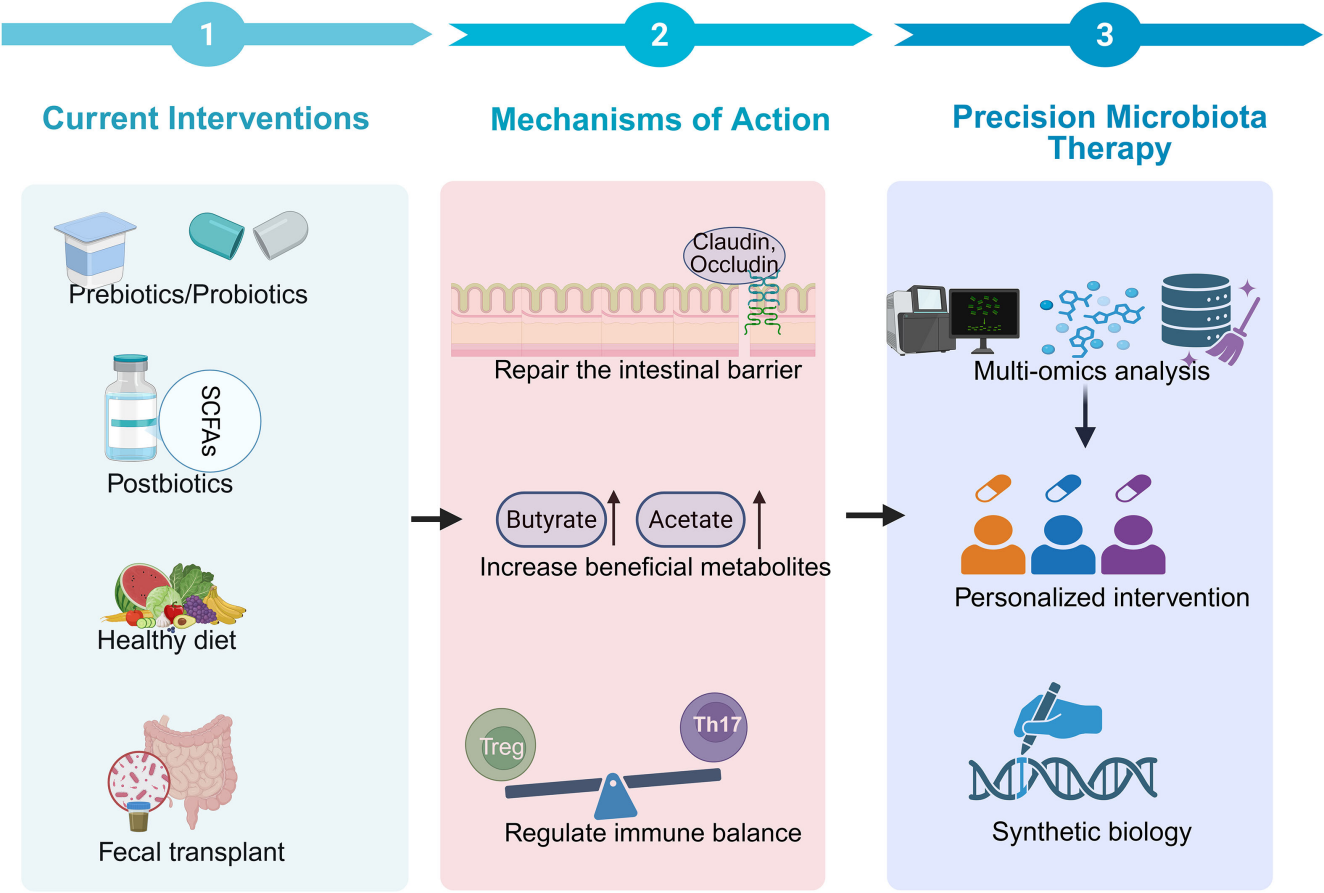
GM regulates bone metabolism through vitamin absorption and mineral homeostasis. Created in Biorender.com.

Future research ought to be directed at employing multi-omics techniques and the use of germ-free animal models. This should be done with the view to achieving mechanistic insights on specific causative pathways, while well-structured prospective clinical trials should be carried out to establish long-term effectiveness and safety. In the meantime, additional inquiries into the use of emerging technologies, such as synthetic biology and nanotechnology, in this area would make “precision microecological treatment” from a mere idea become a well-working practice, thus widening the scope of OP prevention and treatment. A synthesis of the roadmap strategies, which are processed GM-targeted approaches for OP therapy, is illustrated in **Figure 6**.

## 13 Future research directions and emerging prospects: the estrogen-gut-brain axis

The GM and bone relationship niche is vast for precision medicine. Although “gut-bone axis” remains the gold standard, future directions need to focus on more complicated multi-systems. A specific and critical trajectory for future research is the “estrogen-gut-brain axis.”

Clinical and preclinical studies are being conducted to show that estrogen deficiency not only affects the gut and skeleton but also distorts the three important network constellations of the organism, which include the hypothalamus and the pituitary in the case of PMW, functional hypothalamic amenorrhea, and premature ovarian failure, and so on. Estrogen withdrawal generates a drastic change in the gut microbiome composition, in turn leading to the dysregulation of microbial synthesis and secretion of important neurotransmitters, such as 5-HT and gamma-aminobutyric acid (GABA) (298, 299).

Mechanistically, gut-origin 5-HT (synthesized via microbial modulation of TPH-1) is a well-known negative regulator of osteoblast proliferation. Recently, ground-breaking studies conducted in OVX models show proof-of-principle that microbiome-targeted therapeutic modalities can be leveraged to successfully inhibit peripheral 5HT biosynthesis and ameliorate estrogen-deficiency-induced bone loss, along with concomitant modulation of neurobehavioral functions (300, 301).

Deciphering this trivariate neuroendocrine interaction will be pivotal. The complex interplay of estrogen receptors, gut-derived neurometabolites, and central neural circuits in coordinating skeletal muscle mass might represent a new frontier for central-peripheral neuroendocrine effectors. This paradigm shift will open the door to microbiota-specific therapies—including psychobiotics or neuro-modulatory prebiotics working at the nexus of gut-brain-skeleton—to rectify post-menopausal osteoporosis through improved neuropsychiatric outcomes.
